# CD24a knockout results in an enhanced macrophage- and CD8⁺ T cell-mediated anti-tumor immune responses in tumor microenvironment in a murine triple-negative breast cancer model

**DOI:** 10.1186/s12929-025-01165-3

**Published:** 2025-08-09

**Authors:** Shih-Hsuan Chan, Chin-Yu Lin, Hsuan-Jung Tseng, Lu-Hai Wang

**Affiliations:** 1 School of Chinese Medicine, College of Chinese Medicine, China Medical University, No.91, Hsueh-Shih Road, Taichung, 40402 Taiwan; 2 Chinese Medicine Research Center, China Medical University, Taichung, 40402 Taiwan; 3 Cancer Biology and Precision Therapeutics Center, China Medical University, Taichung, 40402 Taiwan; 4 Department of Biomedical Sciences and Engineering, Tzu Chi University, Hualien, 970374 Taiwan; 5 School of Pharmacy, Tzu Chi University, Hualien, 970374 Taiwan; 6 Graduate Institute of Integrated Medicine, China Medical University, Taichung, 40402 Taiwan

**Keywords:** CD24a, CRISPR/Cas9 knockout, Triple-negative breast cancer, Tumor microenvironment, Macrophages, CD8^+^ T cells

## Abstract

**Background:**

CD24 plays a crucial role not only in promoting tumor progression and metastasis but also in modulating macrophage-mediated anti-tumor immunity. However, its impact on the immune landscape of the tumor microenvironment (TME) remains unexplored. Here, we investigated the role of CD24a, the murine CD24 gene, in tumor progression and TME immune dynamics in a murine triple-negative breast cancer (TNBC) model.

**Methods:**

Clustered Regularly Interspaced Short Palindromic Repeat (CRISPR)/Cas9 knockout technology was employed to generate CD24a knockout in the murine TNBC cell line 4T1. Flow cytometry was utilized to analyze the immune cell populations, including myeloid-derived suppressor cells (MDSCs), natural killer cells, T cells, and macrophages, within tumors, spleens, and bone marrow in the orthotopic mouse 4T1 breast cancer model. Immunofluorescence (IF) staining was used to detect the immune cells in tumor sections. High-speed confocal was used to perform three-dimensional (3D) mapping of immune cells in the 4T1 orthotopic tumors.

**Results:**

Knocking out CD24a significantly reduced tumor growth kinetics and prolonged mouse survival in vivo. Flow cytometry and IF analysis of tumor samples revealed that CD24a loss significantly promoted the infiltration of M1 macrophages and cytotoxic CD8^+^ T cells into the TME while reducing the recruitment and expansion of granulocytic MDSCs (gMDSCs). In vitro coculture experiments showed that CD24a deficiency significantly enhanced macrophage‐mediated phagocytosis and CD8⁺ T cell-mediated cytotoxicity, effects that were partially reversed by re‐expression of CD24a. Moreover, in vivo depletion of macrophages and CD8^+^ T cells reverted the delayed tumor growth caused by CD24a knockout, underscoring their critical role in tumor growth suppression associated with CD24a knockout. 3D mapping of immune cells in the TME confirmed the anti-tumor immune landscape in the CD24a knockout 4T1 tumors. Furthermore, in vitro analysis showed that CD24a loss upregulated macrophage colony-stimulating factor expression while suppressed levels of CXCL1, CXCL5, and CXCL10, chemokines known to recruit gMDSCs, further providing a molecular basis for enhanced macrophage recruitment and diminished gMDSC accumulation.

**Conclusions:**

Our findings suggest that CD24a may regulate immune suppression within the TNBC TME. Targeting CD24a enhances macrophage- and CD8⁺ T cell-mediated anti-tumor immune responses and is associated with a shift in the TME toward a more immunogenic state, thereby suppressing tumor growth. These results may support CD24 as a promising immunotherapeutic target for TNBC.

**Supplementary Information:**

The online version contains supplementary material available at 10.1186/s12929-025-01165-3.

## Background

The CD24 gene encodes a highly glycosylated glycosylphosphatidylinositol anchored membrane protein without a transmembrane domain [1, 2]. CD24 has been shown to play diverse functional roles in immunity, cancer, inflammation, and autoimmune diseases [2]. CD24 is predominantly expressed in B-cell progenitors, with a lesser presence in terminally differentiated B cells. It functions as a costimulatory molecule on activated B cells, facilitating the clonal expansion of CD4 T cells [3–5]. Furthermore, CD24 was identified as an immune modulator to suppress the danger-associated molecular pattern (DAMP)-induced inflammatory signals, which could be lethal during host infection [6]. A previous study has established that the interaction between CD24 and Siglec-10 has the capacity to suppress DAMP-mediated inflammatory responses, contributing to the immunosuppression observed in placental tissues [6]. In addition, CD24 overexpression has been associated with several hallmarks of cancer, including increased cell proliferation, migration, and metastasis [3, 7]. CD24's interaction with P-selectin has been demonstrated to facilitate cancer cell adhesion to endothelial cells, promoting pulmonary metastasis [8, 9]. In syngeneic rat models of 1AS pancreatic carcinoma and MTLy mammary carcinoma, ectopic CD24 expression accelerates both primary tumor growth and metastatic dissemination [10]. Additionally, CD24 serves as an oncogenic signaling activator in enhancing pathways including lipid raft/integrin-induced Src signaling [11], the wingless-related integrated site (Wnt)/β-catenin pathway [12], and the Epidermal Growth Factor Receptor (EGFR)/MET proto-oncogene (MET)/protein kinase B (PKB)/extracellular signal-related kinase (Erk) 1/2 pathway [7]. Our previous work on triple-negative breast cancer (TNBC) revealed the involvement of CD24 in TNBC progression and metastatic lung colonization via enhancing anoikis resistance and angiogenesis through receptor tyrosine kinase (RTK) oncogenic pathways such as EGFR and MET [13]. A number of studies have shown that CD24 is an unfavorable marker in the Aldehyde Dehydrogenase 1 (ALDH1)-positive CD44^+^/CD24^−^ breast cancer-initiating cells in human [2, 14, 15]. On the other hand, several studies have identified CD24 as an ovarian cancer stem cell marker, which is pivotal in ovarian cancer progression and metastasis [16, 17]. In the murine 4T1 model, both CD44^+^CD24^−^ and CD44^+^CD24^+^ populations have been positively associated with cancer-initiation potential, illustrating model-specific variability in CD24’s function [10, 18].

In addition to the role of CD24 in cancer progression, recent research has shed light on the function of CD24 as a “don’t eat me” signaling molecule that interacts with macrophage Siglec-10, triggering a signaling cascade that inhibits macrophage-mediated phagocytosis [19]. This "don't eat me" signal is crucial for tumor cells to evade macrophage-mediated anti-tumor immunity, potentially promoting an immunosuppressive tumor microenvironment (TME). However, the direct impact of CD24 loss on tumor cell susceptibility to immune effectors such as macrophages, cytotoxic T cells, and Natural Killer (NK) cells, as well as its specific contribution to establishing an immunosuppressive TME remains unexplored and requires further investigation.

In this study, we employed the Clustered Regularly Interspaced Short Palindromic Repeat (CRISPR)/Cas9 genome-editing technique to knock out the *Cd24a* gene in 4T1 cells, a murine cell line of TNBC, and this allowed us to investigate the role of CD24a in tumor-immune dynamics. We first examined how CD24a loss affects tumor cell sensitivity to immune effectors, including macrophages, CD8^+^ T cells and NK cells in vitro. Subsequently, we assessed the impact of CD24a loss on tumor growth, metastasis, and survival alongside shifts in the TME immune landscape in an orthotopic 4T1/BALB/c mouse model,

## Materials and methods

### Cell lines and reagents

Murine breast cancer cell line 4T1 was obtained from ATCC (American Type Culture Collection) (https://www.atcc.org). 4T1 cells were cultured in Dulbecco’s Modified Eagle Medium (DMEM) supplemented with 10% fetal bovine serum (Life Technologies, Carlsbad, CA, USA) and incubated in a humidified incubator at 37 °C with 5% CO_2_. OPTI-MEM^®^ medium was obtained from Life Technologies. Culture supernatants were sampled and tested for mycoplasma contamination monthly. Cell identity was confirmed via short tandem repeat analysis [20]. Details of the reagents and antibodies used in this study are provided in Additional file 1: Table S1.

### Knockout of Cd24a gene in 4T1 cells using CRISPR/Cas9 technology

Two sgRNAs targeting the upstream of ATG start codon and downstream of exon 1 of *Cd24* gene on mouse genomic DNA were synthesized and cloned into an All-in-one system sgRNA/Cas9 expression lentivector (RNA Technology Platform and Gene Manipulation Core, Taipei, Taiwan), using BsmbI restriction enzyme site. The sequences of sgRNAs were as follows: *Cd24a* sgRNA-1: AGAGTCGCGCCGCGCGCCGA and *Cd24a* sgRNA-2: GGCACTGCTCCTACCCACGC. Two sgRNA/Cas9 expression lentivectors were transiently co-transfected into 4T1 cells using Mirus® XT transfection reagent (Mirus Bio, Madison, WI, USA) for 8 h followed by puromycin selection to remove the untransfected cells. The surviving colonies were subjected to single-cell isolation using limiting dilution, and individual clones derived from single cells were expanded and screened for CD24a expression using flow cytometry. Clones with complete loss of CD24a expression were subjected to genomic DNA extraction. A primer pair was designed to PCR amplify an 804 bp genomic sequence, including 200 bp upstream of the first sgRNA targeting site and 200 bp downstream of the second sgRNA targeting site. Forward primer: 5’-GCCTGTGCATGCAGCCTGC, Reverse primer: 5’-GTGCAGTCGG GAGGTCCCTAA. The PCR products of each positive clone were sequenced using the Sanger sequencing method [21].

### Flow cytometry analysis

To isolate immune cells from tumors, the resected tumors were chopped into pieces and subjected to enzyme digestion using a 1:1 (v/v) mixture of Accutase solution (Innovative Cell Technologies, San Diego, CA, USA) and 0.25% trypsin solution for 1 h at 37 °C as previously described [22]. The digested tumor pieces were passed through a cell strainer, and the tumor-infiltrating cells were further isolated using density gradient centrifugation with Ficoll-Paque^™^ PLUS (Cytiva, Logan, UT, USA). The tumor-infiltrating immune cells were collected from the interface between phosphate-buffered saline (PBS) and Ficoll. For immune cell isolation from mouse spleens [21], the spleens were placed on a cell strainer set on a 50 mL tube and thoroughly crushed with a plunger, followed by washing with 5 mL of PBS to isolate the single-cell population. The tube was centrifuged at 500 g for 5 min, the supernatant was discarded, and the cell pellet was resuspended with red blood cell (RBC) lysis buffer (15.5 mM NH_4_Cl, 1.4 mM NaHCO3, and 50 mM EDTA). This was followed by another centrifugation at 500 g for 5 min to obtain the immune cells. To isolate mouse bone marrow cells, the hind long bones (femora) were dissected, and the ends were cut off with scissors. The bones were flushed with 2 mL of PBS to collect the bone marrow cells, followed by RBC lysis buffer treatment. Next, the samples were centrifuged at 500 g for 5 min to collect bone marrow cells. The populations of CD11b^+^Ly6C^+^ monocytic myeloid-derived suppressor cells (mMDSCs) and CD11b^+^Ly6G^+^ granulocytic MDSCs (gMDSCs) in tumor-infiltrating immune cells, splenocytes, and bone marrow cells were analyzed by staining the cells with fluorescein isothiocyanate (FITC)-conjugated anti-CD11b antibody, PE-Cy7-conjugated anti-Ly6C antibody, and PE-conjugated anti-Ly6G antibody at a concentration of 2 μg/mL. The population of CD11b^+^F4/80^+^ tumor-infiltrating macrophages in tumors was stained with FITC-conjugated anti-CD11b antibody and PerCP-Cy5.5-conjugated F4/80 antibody at a concentration of 2 μg/mL. The population of CD3^+^CD8^+^ tumor-infiltrating T cells in tumors and spleens was analyzed by staining with FITC anti-CD3 antibody, and Per/CP cy5.5-conjuated anti-CD8 antibody at a concentration of 2 μg/mL. The population of CD49^+^ tumor-infiltrating natural killer cells in tumors and spleens was stained with FITC-conjugated anti-CD49b^+^ antibody at a concentration of 2 μg/mL. Fluorescent antibodies used in flow cytometry were purchased from Biolegend Inc. (San Diego, CA, USA). To estimate the absolute numbers of tumor-infiltrating immune cells, we multiplied the total number of immune cells isolated from the tumor with the percentages of the specific immune cell populations determined by flow cytometry analysis. To validate CD24a expression in CD24a knockout (ΔCD24a) 4T1 cells, selected stable clones were first detached from the culture dish using 10 mM EDTA. The detached cells were then stained with 2 μg/mL of PE-conjugated anti-mouse CD24a antibody or isotype control antibody for 30 min, followed by washing with PBS. The isotype control group was used as a fluorescence baseline to set appropriate thresholds for identifying positive populations. Flow cytometry data were acquired using a BD Canto (Becton, Dickinson and Company, Franklin Lakes, NJ, USA), and subsequent analysis was performed using FlowJo^™^ software (Becton, Dickinson and Company).

### Tumor spheroid formation assay

A total of 1 × 10^5^ cells were resuspended and seeded in the ultra-low 6-well plates with MACS sphere medium (Miltenyi Biotec, Inc., Auburn, CA, USA). The cells were cultured for 7 days in a humidified incubator at 37 ℃ supplied with 5% CO_2_. The images of tumor spheroids were photographed using light microscopy and the number and size of spheroids were quantified using Image J software.

### MTS metabolic assay

3 × 10^3^ 4T1 cells and ΔCD24a 4T1 cells were seeded in a 96-well plate respectively 24 h prior to the experiment. The metabolic activity of cells was then evaluated using CellTiter 96^®^ AQueous One Solution Cell Proliferation (MTS) Assay (Promega, Madison, WI, USA). The detailed procedure was described elsewhere [23].

### Cell proliferation assay

Briefly, 8,000 cells per well were seeded in triplicate for each treatment condition into 96-well plates on day 0. Cells were incubated at 37 °C with 5% CO₂, and the number of viable cells was determined daily from day 1 to day 4 using a hemocytometer and trypan blue exclusion to distinguish live from dead cells. Cell proliferation curves were plotted using GraphPad Prism 9.0 software.

### RNA extraction and reverse transcription-quantitative polymerase chain reaction

Cell samples were harvested and lysed in 1 mL of TRIzol reagent (Invitrogen, Carlsbad, CA, USA) to extract RNA. To separate the phases, 200 µL of chloroform was added to the lysate, followed by vigorous shaking for complete mixing. The mixture was incubated on ice for 5 min before centrifugation, which separated the aqueous (RNA-containing) from the organic phase. The aqueous phase was carefully transferred to a clean tube. To precipitate RNA, an equal volume of isopropanol was added, mixed gently, and incubated on ice for 30 min. The mixture was then centrifuged to pellet the RNA, and the supernatant was discarded. The RNA pellet was washed with 75% ethanol to remove impurities and then dissolved in RNase-free water. For reverse transcription, 1 µg of RNA was converted into complementary DNA using Superscript reverse transcriptase according to the manufacturer's instructions (Invitrogen). Quantitative PCR (qPCR) was performed in QuantStudio Real-Time PCR systems (Thermo Fisher Scientific Inc., St. Louis, MO, USA) using gene-specific primers and KAPA SYBR® FAST qPCR Master Mix (Roche, Indianapolis, IN, USA), with the following cycling conditions: Initial denaturation at 95 °C for 5 min, followed by 40 cycles of 95 °C for 15 s and 60 °C for 30 s. Gene expression levels were analyzed using the ΔΔCT method, where ΔΔCT represents the difference in threshold cycle (CT) values between the experimental and control samples, calculated as 2^(−ΔΔCT)^. Primers used for the amplification of *Ccl2, Ccl5, Csf1, Cxcl3, Cxcl5, Cxcl10* and *Cxcl16* were detailed in Additional file 1: Table S2.

### Exon array analysis

RNA was extracted from 4T1 cells and ΔCD24a 4T1 cells and then analyzed using the Affymetrix GeneChip Human Exon 1.0 ST Array (Affymetrix, Santa Clara, CA, USA) for transcriptome analysis. All exon array data was analyzed using tools in Transcriptome Analysis Console (TAC) Software. (Thermo Fisher Scientific, St. Louis, MO, USA).

### Animal study

All procedures were performed in accordance with institutional and national guidelines for the care and use of laboratory animals. BALB/c ByJNarl (B/c) female mice (6 weeks old) were obtained from the National Laboratory Animal Center, Taiwan. Mice were housed in individually ventilated cages under specific pathogen-free conditions with a 12-h light/dark cycle, controlled temperature (22 ± 2 °C), and humidity (50%–60%), with ad libitum access to autoclaved food and water. Environmental enrichment, including nesting material and shelters, was provided. For tumor implantation, 2 × 10^4^ 4T1 or ΔCD24a 4T1 cells were suspended in PBS and mixed with Matrigel (Corning, Glendale, AZ, USA) at a 2:1 (v/v) ratio. Cell suspensions (final volume: 100 µL) were injected orthotopically into the fourth mammary fat pad of anesthetized mice (anesthesia method: isoflurane). Five mice were assigned to each group (4T1 and ΔCD24a 4T1). Tumor growth was measured weekly using calipers, and tumor volume was calculated using the formula: (length × width^2^)/2. Mice were monitored at least twice weekly for signs of morbidity. On day 34 post-inoculation, mice were euthanized via CO₂ inhalation. Tumors were excised into two portions: one was fixed in 2% paraformaldehyde, embedded in tissue freezing medium (Leica Microsystems, Wetzlar, Germany), and stored at −80 °C for subsequent immunofluorescence (IF) staining, while the other portion was processed fresh for flow cytometry analysis of immune cell populations. For the survival study, an independent cohort of mice (n = 5 per group) was similarly implanted with 2 × 10^4^ 4T1 or ΔCD24a 4T1 cells. Mice were monitored daily for survival for up to 80 days post-implantation. Humane endpoints were defined as tumor diameter exceeding 2 cm, severe ulceration, significant weight loss (> 20%), or signs of distress. Mice reaching humane endpoints were immediately euthanized. Animals were randomized into experimental groups, and investigators were blinded to group allocations during outcome assessments. Sample size was determined based on prior experience with the 4T1 model and statistical power considerations to detect meaningful biological differences.

### Cell cycle and sub G1 phase analysis

Cells were detached using trypsin–EDTA, combined with the floating cells, and washed twice with cold PBS. Cells were then fixed in 70% ethanol at −20 °C for at least 2 h. After fixation, cells were washed with PBS and incubated with a staining solution containing 50 μg/mL propidium iodide and 100 μg/mL RNase A (Sigma-Aldrich, St. Louis, MO, USA) in PBS for 30 min at room temperature in the dark to allow for RNA digestion. DNA content was analyzed using a flow cytometer BD Canto (Becton, Dickinson and Company), and the percentage of cells in the sub-G₁ population, representing apoptotic cells, was quantified by gating.

### Western blot analysis

Cells were lysed in RIPA buffer supplemented with protease and phosphatase inhibitors (Sigma-Aldrich). Protein concentrations were determined using the BCA assay (Thermo Fisher Scientific, Waltham, MA, USA). A total of 20 µg of protein per sample were resolved by SDS-PAGE and transferred onto PVDF membranes (Millipore, Burlington, MA, USA). Membranes were blocked with 5% non-fat dry milk in TBST (Tris-buffered saline containing 0.1% Tween-20) for 1 h at room temperature, followed by overnight incubation at 4 °C with the following primary antibodies at a concentration of 0.2 µg/mL in TBST: anti-EGFR antibody (#4267, Cell Signaling Technology, Danvers, MA, USA), anti-phospho-EGFR antibody (#3777, Cell Signaling Technology), and anti-GAPDH antibody (#5174, Cell Signaling Technology) as a loading control. After washing, membranes were incubated with HRP-conjugated secondary antibodies (Cell Signaling Technology) at a concentration of 0.04 µg/mL in TBST for 1 h at room temperature. Bands were visualized using enhanced chemiluminescence (ECL) reagents (Thermo Fisher Scientific) and imaged with a ChemiDoc™ Imaging System (Bio-Rad, Hercules, CA, USA).

### IF staining

The frozen mouse tumors were sectioned into 10 μm tissue slices using a freezing microtome. Briefly, the tumor slices were washed with PBS three times and then were blocked in the blocking buffer (PBS containing 5% goat serum and 1% bovine serum albumin) for 1 h at room temperature. The tumor sections were washed with PBS to remove the blocking buffer followed by incubation of the specific primary antibodies at a concentration of 2 μg/mL overnight at 4℃. The tumor sections were washed with PBS three times to remove the primary antibodies followed by incubation of the fluorescence-tagged secondary antibodies for 1 h at room temperature. The tumor slices were washed with PBS buffer and mounted with anti-fade mounting medium containing DAPI (4',6-diamidino-2-phenylindole) (Life Technologies). Detailed information on the IF primary antibodies, including the anti-F4/80 antibody, anti-CD206 antibody, anti-CD86 antibody, anti-CD8α antibody, and anti-Gr-1 antibody, is provided in Additional file 1: Table S1.

### Three-dimensional (3D) imaging

Mouse tumors were fixed in 4% paraformaldehyde and sectioned into 500 μm slices using a 5100mz vibrating microtome (Campden Instruments Ltd., Loughborough, Leicestershire, UK). Following fixation, the sections were washed three times with PBS. The tumor sections were then permeabilized with 2% Triton X-100 overnight, followed by three additional PBS washings to remove Triton-X100. The tumor slices were blocked overnight in a blocking buffer containing 10% goat serum in PBS. Primary antibodies were prepared at a concentration of 2 μg/mL in an antibody dilution buffer (PBS containing 1% goat serum and 0.25% Triton-X100) and incubated with the tumor sections for 48 h at 4 ℃. Afterward, the sections were washed three times by a washing buffer (0.1% Triton-X100 in PBS) at 4 ℃. The tumor slices were then incubated with the fluorescence-conjugated secondary antibodies (1 μg/mL in the antibody dilution buffer) for 24 h at 4 ℃ followed by three PBS washes in a washing buffer at 4 ℃. Next, the tumor slices were stained with Hoechst (1:500 dilution) for 15 min followed by PBS wash three times at room temperature to remove Triton-X100. Finally, the tumor sections were treated with RapiClear CS solution (SunJin Lab Co., Hsinchu City, Taiwan) at room temperature for 1 h to achieve the tissue clearing. The 3D image of the tumor sections was obtained using the ANDOR Dragonfly High Speed Confocal (Oxford Instruments, Abingdon, Oxfordshire, UK), and the images were analyzed and generated using Imaris 9.7 image analysis software.

### Isolation of CD8^+^ T cells and CD49b^+^ natural killer cells from mouse spleens

Mouse spleens were harvested and mechanically disrupted using a cell strainer to release splenocytes containing CD8^+^ T cells and CD49b^+^ NK cells. The collected splenocytes were suspended in PBS and centrifuged at 500 g for 5 min at room temperature to pellet the cells. The supernatant was discarded, and the cell pellet was resuspended in 2–5 mL of cold RBC lysis buffer. The suspension was incubated for 4–5 min on ice to lyse erythrocytes. After lysis, the cell suspension was washed with 10–20 mL of cold PBS and centrifuged at 500 g for 5 min at 4 °C; the supernatant was discarded. The cell pellet was then resuspended in PBS. Subsequently, CD8^+^ T cells and CD49b^+^ NK cells were isolated using the EasySep™ Mouse CD8 + T Cell Isolation Kit and EasySep^™^ Mouse NK Cell Isolation Kit (Stem cell Technologies, Vancouver, Canada), respectively, according to the manufacturer’s protocols.

### Preparation of bone marrow-derived macrophages (BMDMs) and M1 polarization

Bone marrow cells were similarly isolated as described above. The isolated bone marrow cells were resuspended in complete RPMI-1640 medium supplemented with 100 ng/mL macrophage colony-stimulating factor (M-CSF) (Stem cell Technologies, Vancouver, Canada). Cells were plated at a density of 1 × 10^6^ cells/mL in Petri dishes and incubated at 37 °C with 5% CO₂. On day 3, half of the medium was replaced with fresh M-CSF-containing medium, and by day 7, adherent cells exhibited macrophage morphology and expressed macrophage-specific markers F4/80 and CD11b. To induce M1 polarization, BMDMs were further stimulated with 100 ng/mL IFN-γ (Stem cell Technologies, Vancouver, Canada), for 24 h. Following stimulation, M1 macrophage differentiation was confirmed by assessing expression of M1 lineage markers CD11b, F4/80 and CD80 using flow cytometry. The polarized M1 macrophages were subsequently used for in vitro phagocytosis.

### In vitro phagocytosis

4T1 and ΔCD24a 4T1 cells were harvested and labeled with 1 µM Calcein-AM (Thermo Fisher Scientific Inc., Waltham, MA, USA) according to the manufacturer’s instructions. Labeled cells were washed twice with PBS to remove excess dye. M1 macrophages were seeded in 24-well plates at a density of 0.5 × 10^5^ cells per well and allowed to adhere overnight. 2 × 10^5^ cells 1abeled 4T1 and ΔCD24a 4T1 cells were then added to the wells. The co-cultures were incubated at 37 °C with 5% CO_2_ for 4 h. After the incubation, engulfed cancer cells were observed under a fluorescence microscope, and phagocytosis was quantified by determining the percentage of macrophages exhibiting Calcein-AM fluorescence, indicating the engulfment of labeled cancer cells.

### In vitro cytotoxicity assay

Briefly, 1.5 × 10^4^ 4T1 or ΔCD24a 4T1 cells were seeded into 96-well plates and incubated overnight at 37 °C in a 5% CO_2_ incubator. CD49b^+^ NK cells and CD8^+^ T cells were isolated from the spleens of BALB/c mice using the EasySep™ Mouse NK Cell Isolation Kit and EasySep^™^ Mouse CD8 + T Cell Isolation Kit (Stem cell technologies, Vancouver, Canada), and added to the target cells at an effector-to-target (E:T) ratio of 20:1. The co-cultures were incubated for 48 h, and cell viability was assessed by the trypan blue exclusion assay. The number of viable (unstained) and dead (blue-stained) cells was counted using a hemocytometer. Cytotoxicity was calculated by comparing the viability of target cells in the co-culture condition to that of target cells cultured alone, using the formula: Cytotoxicity (%) = (Control Viability—Experimental Viability) / Control Viability) × 100. This assay was performed for both 4T1 and ΔCD24a 4T1 cells to evaluate the extent of cytotoxicity mediated by CD49b^+^ NK cells and CD8^+^ T cells.

### Gene correlation analysis

The cBioportal (https://www.cbioportal.org) was utilized to access the TCGA (The Cancer Genome Atlas) Breast Invasive Carcinoma dataset. Normalized mRNA expression z-scores for the genes were retrieved from 1084 patients, and Spearman correlation analysis of the relevant genes was conducted using GraphPad Prism 9.0 software.

### Statistical analysis

Statistical analyses were conducted using methods appropriate for the data and study objectives. Differences between two groups were assessed by unpaired, two‐tailed Student’s t-test. Comparisons among three or more groups were made by one‐way ANOVA followed by Tukey’s multiple‐comparisons test. Kaplan–Meier survival curves were compared using the log-rank test to evaluate differences in survival distributions. The Cox proportional hazards model was used to estimate hazard ratios between groups. Statistical significance was defined as a P < 0.05 for all analyses.

## Results

### The *Cd24a* gene was successfully knocked out in 4T1 murine breast cancer cells using the CRISPR/Cas9 approach

To establish ΔCD24a 4T1 cells via the CRISPR/Cas9 method, we designed and inserted two sgRNAs downstream of the U6 promoter in Cas9-expressing plasmids. Specifically, one sgRNA targeted sequences upstream of the transcription start site of the *Cd24a* gene, while the other targeted the exon 1-intron 1 junction of the *Cd24a* gene in the mouse genome. 4T1 cells were transfected with sgRNA-containing Cas9-expressing plasmids followed by puromycin selection. The *Cd24a* gene knockout status of the resulting stable cells was validated by Sanger sequencing, which revealed a successful deletion of exon 1 (Additional file 2: Fig. S1A) when the sequencing data was mapped to mouse genome using the UCSC (University of California Santa Cruz) Genome Browser (https://genome.ucsc.edu). This deletion was further validated by RNA sequencing, as indicated by the absence of exon 1 (Additional file 2: Fig. S1B). Flow cytometry was used to further validate the loss of membrane CD24a expression in two ΔCD24a 4T1 clones, ΔCD24a-1 and ΔCD24a-2, as compared to WT 4T1 cells (Additional file 2: Fig. S1C).

### CD24a knockout enhanced tumor cell sensitivity to immune effectors, macrophages and CD8^+^ T cells

**Fig. 1:**
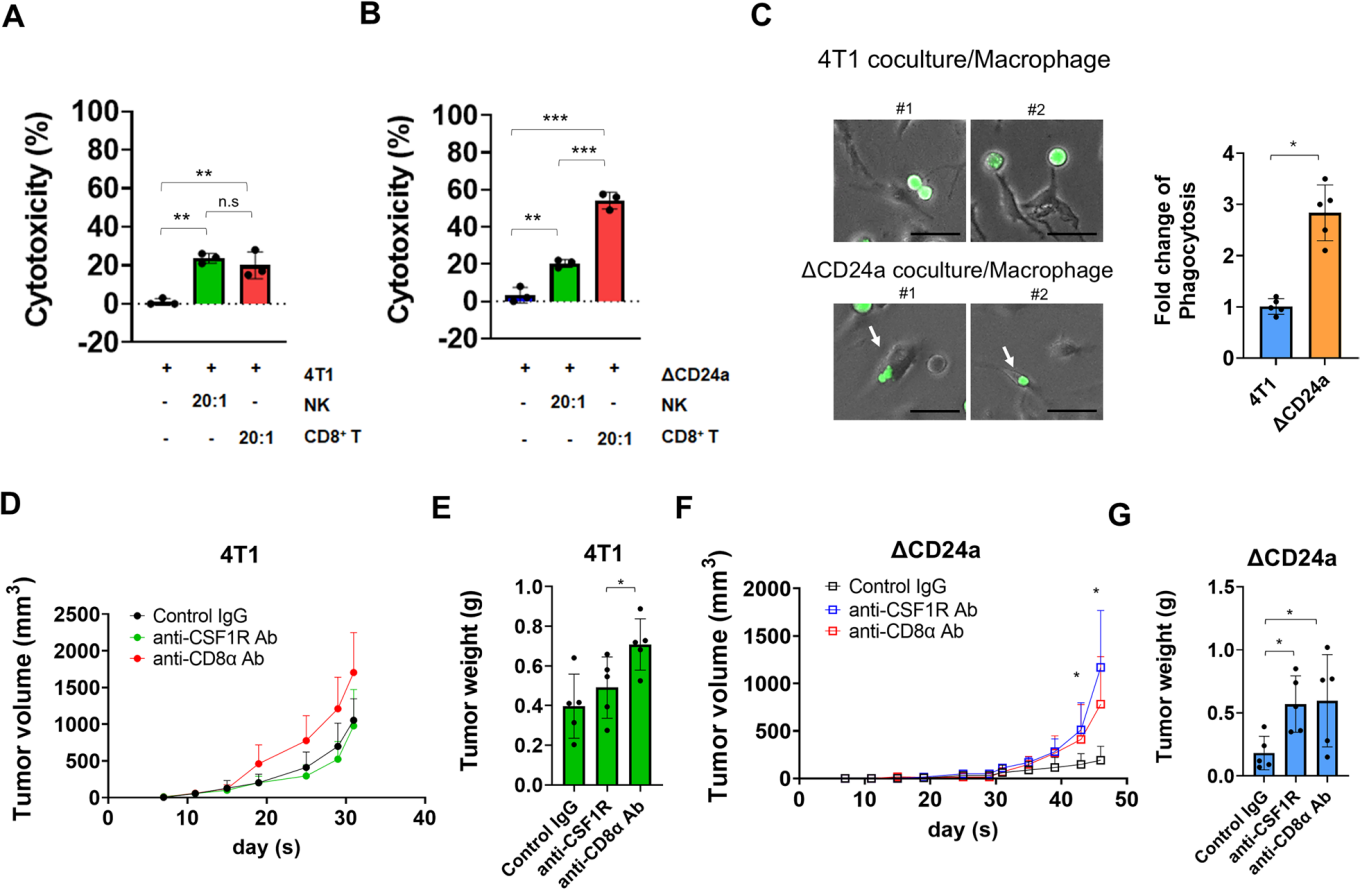
Depletion of macrophages and CD8^+^ T cells abolishes CD24a knockout-induced growth delay of 4T1 tumors. **A** The cytotoxicity effect of NK cells and CD8^+^ T cells on 4T1 cells. NK cells and CD8^+^ T cells were isolated from mouse spleen and purified as described in the Methods section 1.5 × 10^4^ 4T1 cells were incubated with NK cells and CD8^+^ T cells at an effector-to-target (E:T) ratio of 20:1 for 48 h. After incubation, cell viability was assessed using trypan blue exclusion staining to determine the extent of cytotoxicity. **B** The cytotoxicity effect of NK cells and CD8^+^ T cells on ΔCD24a cells. 1.5 × 10^4^ ΔCD24a 4T1 cells were incubated with NK cells and CD8^+^ T cells at an E:T ratio of 20:1 for 48 h. Cell viability was similarly determined. **P < 0.005, ***P < 0.001. **C** Representative images of in vitro phagocytosis. Cancer cells were pre-labeled with Calcein-AM dye (green fluorescence), followed by coculturing with M1 macrophages for 4 h. M1 macrophages were prepared as described in the Methods section. Phagocytosis analysis was measured by comparing the percentage of cancer cell-engulfing macrophages in the 4T1/M1 macrophages group and ΔCD24a 4T1/macrophage group under a fluorescence microscope (100 × magnification). Scale bar: 40 μm. *P < 0.05. **D** Tumor growth kinetics of 4T1 tumor-bearing mice receiving anti-CD8α, anti-CSF1R, or control IgG (n = 5). **E** Endpoint tumor weight analysis of 4T1 tumor-bearing mice treated with anti-CD8α, anti-CSF1R, or control IgG (n = 5). *P < 0.05. **F** Tumor growth kinetics of ΔCD24a 4T1 tumor-bearing mice receiving anti-CD8α, anti-CSF1R, or control IgG (n = 5). *P < 0.05. **G** Endpoint tumor weight analysis of ΔCD24a 4T1 tumor-bearing mice treated with anti-CD8α, anti-CSF1R or control IgG (n = 5). *P < 0.05

To investigate the direct effects of CD24a loss on tumor cell-immune cell interactions, we first examined the susceptibility of ΔCD24a 4T1 cells to immune effectors, including macrophages, cytotoxic T cell, and NK cells in vitro. In in vitro cytotoxicity experiments, when co-cultured with either CD49b^+^ NK cells or CD8^+^ T cells at an effector-to-target (E:T) ratio of 20:1, 4T1 cells exhibited similar susceptibility to cytotoxicity (Fig. 1A). In contrast, co-culture of ΔCD24a 4T1 cells with CD8^+^ T cells resulted in a significant 30% higher in tumor cell cytotoxicity than co-culture with CD49b^+^NK cells. (Fig. 1B). To explore the effect of CD24a knockout on macrophage activity, M1 macrophages were prepared as described in the Methods section. Successful M1 polarization was confirmed by the expression of lineage-specific markers, including CD11b, F4/80, and CD80, as verified by flow cytometry (Additional file 3: Fig. S2). The result of in vitro phagocytosis assay demonstrated that M1 macrophages exhibited significantly increased phagocytic activity towards ΔCD24a cells compared to 4T1 cells (Fig. 1C). Moreover, re-expression of CD24a in ΔCD24a 4T1 cells significantly restored resistance to macrophage-mediated phagocytosis and CD8⁺ T cell-mediated cytotoxicity (Additional file 4: Fig. S3A and S3B).

To further elucidate the roles of CD8^+^ T cells and macrophages in vivo, we conducted immune cell depletion experiments in tumor-bearing mice. In 4T1 tumor-bearing mice, depletion of CD8^+^ T cells resulted in a marked acceleration of tumor growth (Fig. 1D, E), whereas depletion of macrophages showed no significant impact on primary tumor growth (Fig. 1D, E). In contrast, depletion of CD8^+^ T cells and macrophages both resulted in a significant increase of tumor growth in ΔCD24a 4T1 tumor-bearing mice (Fig. 1F, G). Our results indicate that CD24a knockout in 4T1 cells enhances anti-tumor immune response by increasing the abundance of immune effectors in TME and increasing tumor cell susceptibility to CD8^+^ T cells-mediated cytotoxicity and macrophages-mediated phagocytosis. While CD8^+^ T cell depletion accelerates tumor growth in both 4T1 and ΔCD24a tumor models, macrophage depletion only impacts tumor growth in the ΔCD24a model, suggesting a pivotal role of CD24a in macrophage-driven anti-tumor immunity.

### CD24a knockout impaired the tumorigenic potential of 4T1 cells

**Fig. 2:**
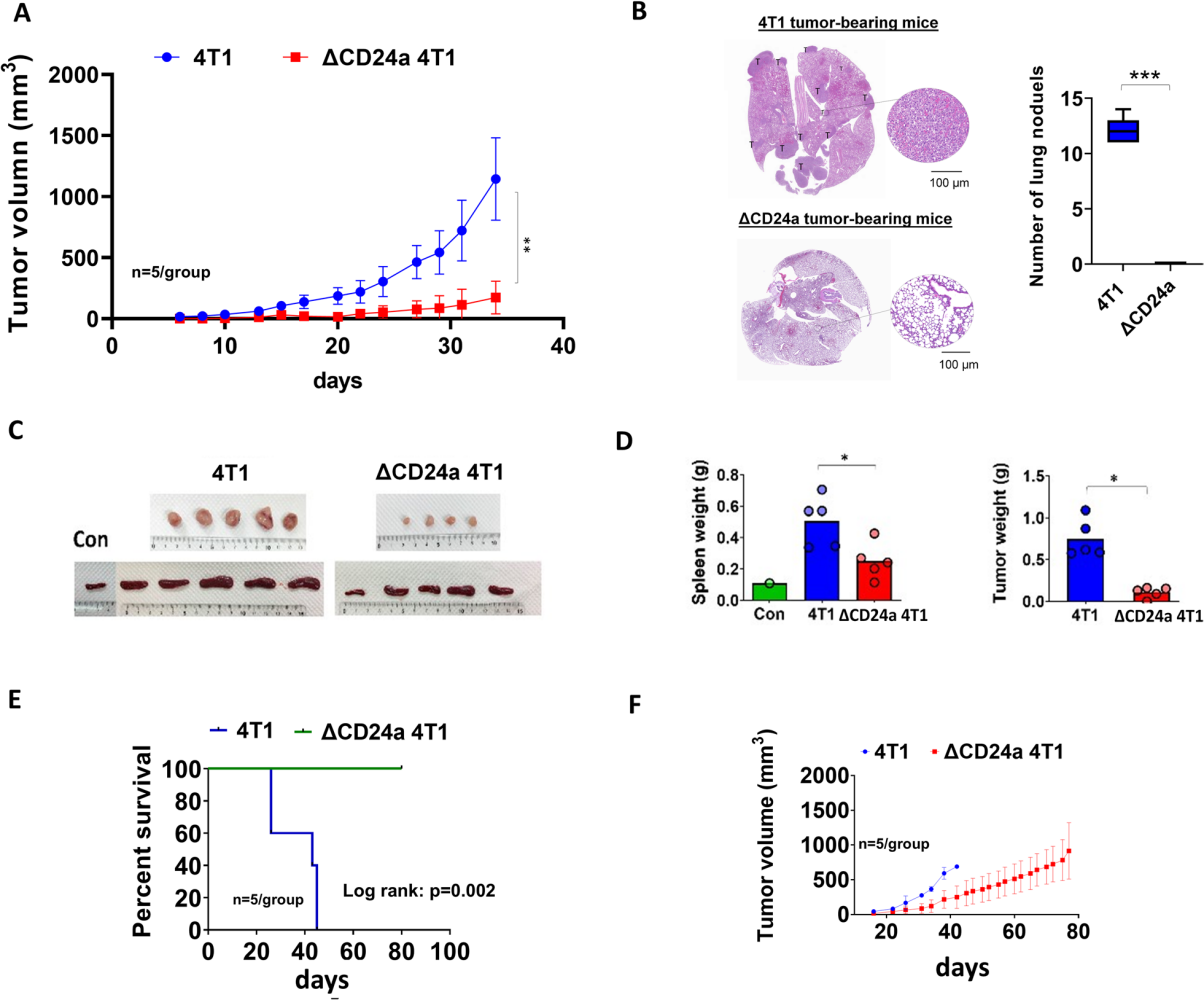
Knockout of CD24a significantly impairs tumor growth and prolongs the mouse survival in a 4T1 BALB/c syngeneic model. **A** Tumor growth kinetics of ΔCD24a 4T1 tumor-bearing (n = 5) and WT 4T1-bearing mice (n = 5). The abscissa shows days after tumor cell injection. **P < 0.01. **B** Lung metastasis analysis in 4T1 and ΔCD24a 4T1 tumor-bearing mice. Left Panel: Representative H&E staining images of lung sections from 4T1 and ΔCD24a 4T1 tumor-bearing mice, showing metastatic nodules (labeled "T"). Right Panel: Quantification of lung metastatic nodules in 4T1 and ΔCD24a 4T1 tumor-bearing mice. **C** Images of resected tumors and spleens from ΔCD24a 4T1 tumor-bearing (n = 5) and 4T1 tumor-bearing mice (n = 5). Spleens resected from tumor-free mice were also shown. **D** The weight of tumors and spleens are shown in histograms. **E** Kaplan–Meier survival analysis of ΔCD24a 4T1 tumor-bearing mice and 4T1 tumor-bearing mice. Log rank test: P = 0.002. **F** Tumor growth kinetics of ΔCD24a 4T1 tumor-bearing (n = 5) and 4T1 tumor-bearing mice (n = 5) during the survival experiment. All ΔCD24a 4T1 tumor-bearing survived to the endpoint of the experiment

We first examined the effect of CD24a knockout on spheroid-forming ability of 4T1 cells to evaluate the capacity of 4T1 and ΔCD24a 4T1 cells to form 3D spheroids in vitro. We found that CD24a knockout significantly impaired spheroid-forming ability of 4T1 cells (Additional file 5: Fig. S4A) without affecting cell metabolic activity (Additional file 5: Fig. S4B). Both the number and size of spheroids were markedly decreased in the ΔCD24a 4T1 cells. (Additional file 5: Fig. S4A). Spheroid formation has been linked to aggressive characteristics of cancer cells but does not directly measure tumor initiation potential in vivo. We previously showed that depletion of CD24 enhanced the degradation of RTK such as EGFR, thereby impairing anchorage-independent growth in human TNBC cells [13]. We next investigated whether CD24a loss similarly affected EGFR expression and activation in the 4T1 model. Consistent with our previous findings, CD24a knockout resulted in reduced EGFR protein levels and diminished EGF-induced EGFR phosphorylation (Additional file 6: Fig. S5A). Importantly, cell proliferation assay and sub‐G1 apoptosis analyses confirmed that these effects occur independently of changes in basal cell proliferation or survival (Additional file 6: Fig. S5B-S5D). Next, we performed in vivo studies to confirm whether the loss of CD24a affected the tumor growth kinetics of 4T1 cells. Subsequently, ΔCD24a-1 clone was selected for the following in vivo study. In the 4T1/BALB/c syngeneic mouse model, we showed that ΔCD24a 4T1 cells exhibited a significant delay in tumor growth compared to the 4T1 cells (Fig. 2A, C, D). Additionally, CD24a knockout was associated with reduced lung metastasis (Fig. 2B). In addition, ΔCD24a 4T1 tumor-bearing mice showed less splenomegaly than the 4T1 tumor-bearing mice (Fig. 2C, D), and a longer survival time was noted (Fig. 2E, P = 0.002, hazard ratio: 0.049. 95% CI 0.007–0.348). Tumor growth kinetics during the survival experiment are shown in Fig. 2F. When comparing mice with similar primary tumor burdens, ΔCD24a tumor-bearing mice showed a reduced number of lung metastatic nodules compared to 4T1 tumor-bearing mice (Additional file 7: Fig. S6A-S6C). These data suggest that loss of CD24a led to a significant delay in tumor growth in the 4T1/BALB/c syngeneic mouse model. While the tumor growth delay could be partially explained by reduced RTK activity such as EGFR, the role of CD24a in immune modulation prompted us to investigate whether its knockout affects the TME.

### CD24a knockout induced macrophage infiltration into the TME

**Fig. 3:**
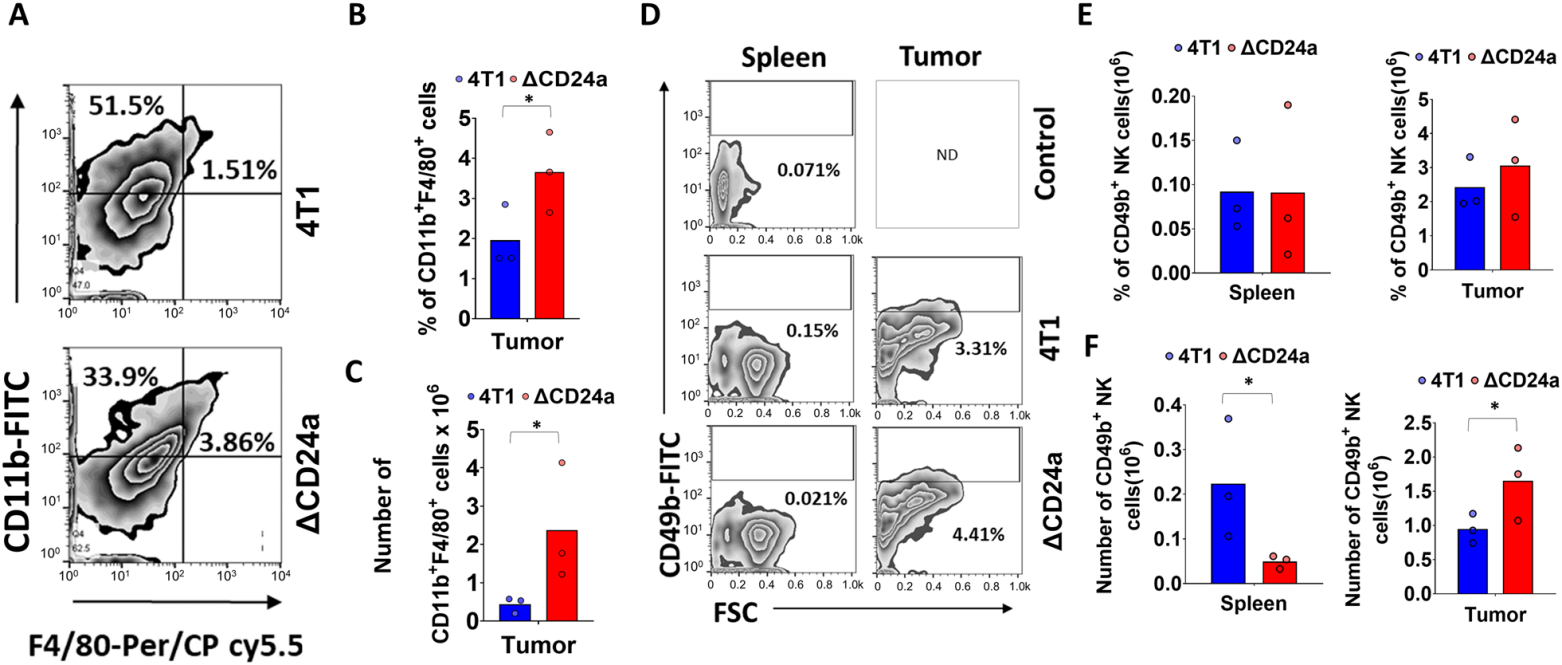
The loss of CD24a significantly enhances macrophage infiltration into the TME. **A** Flow cytometry analysis of tumor-infiltrated CD11b^+^ F4/80^+^ macrophages in the tumors resected from 4T1 tumor-bearing mice and ΔCD24a 4T1 tumor-bearing mice (n = 3). Cells were isolated from tumors using protocol described in the Method section. The isolated cells were stained with FITC-conjugated anti-CD11b antibody and PerCp/Cyanine5.5-conjugated anti-F4/80 antibody, followed by flow cytometry analysis. **B** The percentages and **C** numbers of CD11b^+^F4/80^+^ macrophages in the tumors were analyzed with FlowJo^™^ software and presented in histograms. *P < 0.05. **D** Flow cytometry analysis of the presence of CD49b^+^ NK cells in the tumors and spleens from 4T1 tumor-bearing mice and ΔCD24a 4T1 tumor-bearing mice (n = 3). Cells isolated from the spleens and tumors were stained with FITC-conjugated anti-CD49b antibody. **E** Histograms show mean percentage and **F**, number of CD49b.^+^ NK cells in the spleens and tumors resected from 4T1 tumor-bearing mice and ΔCD24a 4T1 tumor-bearing mice. *P < 0.05

First, we explored the effects of CD24a ablation on the infiltration of tumor-associated macrophages (TAMs), given that CD24 is known to suppress macrophage phagocytic function. Despite this, the mechanistic contributions of CD24 within the TME remain insufficiently elucidated. Using flow cytometry, we analyzed the presence of CD11b^+^/F4/80^+^ TAMs in the ΔCD24a 4T1 and 4T1 tumors. Dot plot analysis revealed a higher percentage of CD11b^+^/F4/80^+^ TAMs in the ΔCD24a 4T1 tumors compared to 4T1 tumors (Fig 3A). Histogram analysis showed a more abundance (over 80%) of TAMs in the ΔCD24a 4T1 tumors compared to that in 4T1 tumors (Fig. 3B, n=3). Moreover, the number of TAMs in the ΔCD24a 4T1 tumors demonstrated more than a two-fold increase relative to the 4T1 tumors (Fig. 3C), indicating a substantial elevation in TAM infiltration in the ΔCD24a 4T1 tumors. We subsequently examined NK cell distribution in the spleens and tumors of tumor-bearing mice. Dot plot analysis of flow cytometry showed that ΔCD24a 4T1 tumors-bearing mice showed similar percentage of CD49b^+^ NK cells in the spleens and a non-significant increase in the tumors compared to 4T1 tumors-bearing mice (Fig. 3D). Histogram analysis showed comparable mean percentage of splenic CD49b^+^ NK cells for three mice in each group (Fig. 3E). However, ΔCD24a 4T1 tumor-bearing mice had fewer CD49b^+^ NK cells in the spleens but a greater number in the tumors compared to 4T1 tumor-bearing mice (Fig. 3F). These findings indicate that CD24a ablation substantially enhances TAM infiltration into the TME, both in percentage and number, suggesting a potential role for macrophages in the observed tumor shrinkage.

### CD24a knockout significantly promoted the infiltration of cytotoxic CD8^+^ T cells into the TME

**Fig. 4:**
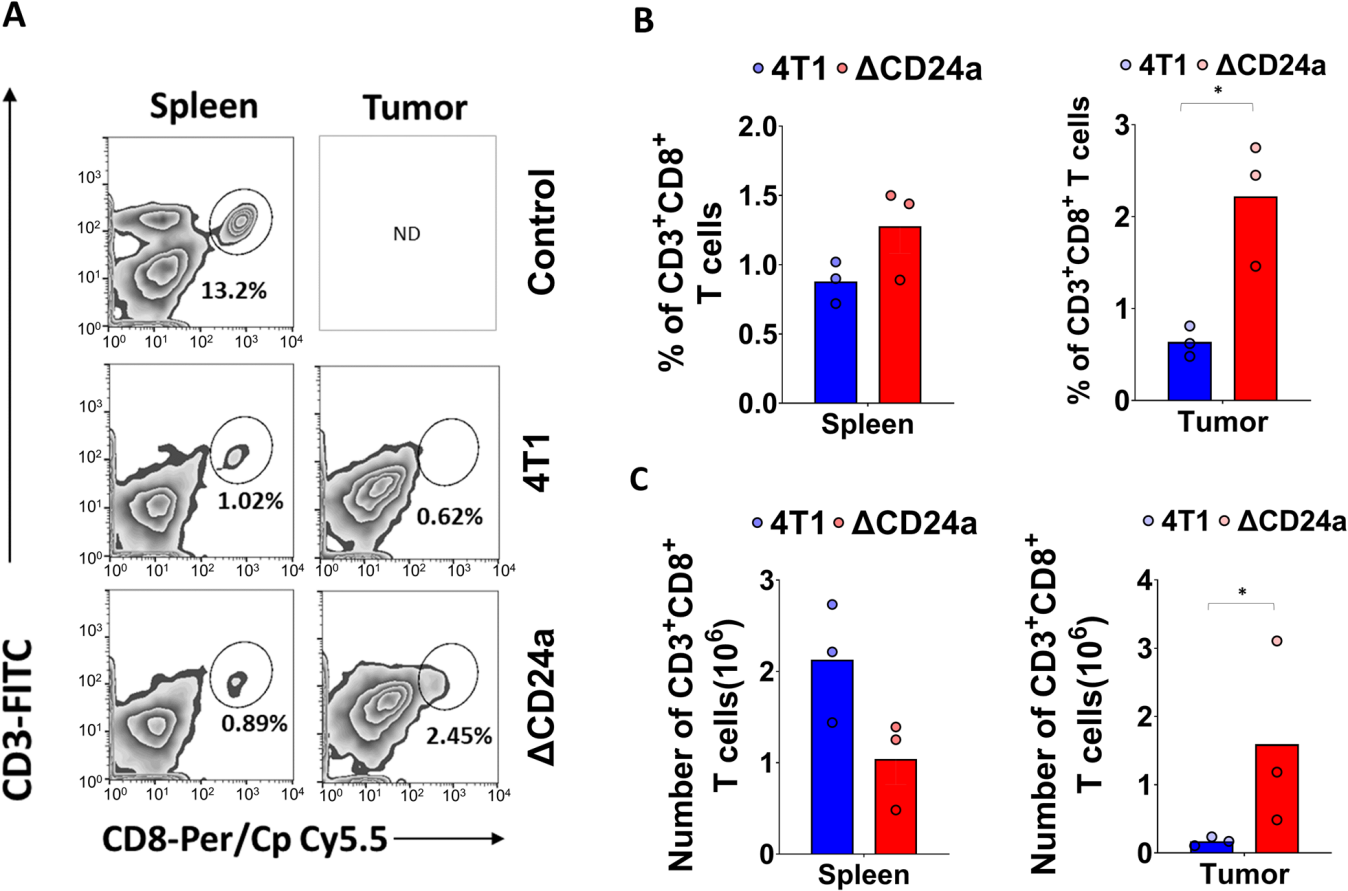
CD24a loss significantly enhances CD8^+^ T cells infiltration into tumors. **A** Flow cytometry was used to analyze CD8⁺ T cell infiltration in the spleens and tumors resected from 4T1 tumor-bearing mice and ΔCD24a 4T1 tumor-bearing mice (n = 3). Cells were isolated from spleens and tumors using the protocol described in the Method section. The isolated cells were stained with FITC-conjugated anti-CD3 antibody and PerCp/Cyanine5.5-conjugated anti-CD8 antibody, followed by flow cytometry analysis. **B** Histograms show mean percentage and **C** cell number of CD8^+^ T cells in the spleens and tumors resected from 4T1 tumor-bearing mice and ΔCD24a 4T1 tumor-bearing mice. *P < 0.05

Given the reduced tumor burden observed in ΔCD24a tumor-bearing mice compared to the 4T1 tumor-bearing mice, we explored the effects of CD24a ablation on cytotoxic CD8^+^ T cell infiltration into the TME, a critical mechanism in anti-tumor immunity. As depicted in the Fig. 4A, dot plot analysis revealed a marked elevation in the proportion of CD3^+^CD8^+^ T cells within the ΔCD24a tumors compared to that in the 4T1 tumors. Histogram analysis displaying the mean percentage and cell count of CD3^+^CD8^+^ T cells from three mice in each group indicated a significantly more infiltrated CD8^+^ T cells in the TME of ΔCD24a relative to that of 4T1 tumors (Fig. 4B, C). In contrast, ΔCD24a 4T1 tumor-bearing mice exhibited a comparable percentage, yet a reduced abundance of CD8^+^ T cells in the spleen (Fig. 4B, C), potentially associated with the smaller spleen size observed in these mice compared to the mice bearing 4T1 tumors (Fig. 2D). Collectively, these findings suggest that CD24a knockout in tumor cells significantly promotes the infiltration of cytotoxic CD8^+^ T cells into the tumors.

### CD24a knockout significantly suppressed the recruitment of gMDSCs to the TME

We next investigated the effect of CD24a ablation on the distribution of MDSCs, a heterogeneous population of immature myeloid cells known to accumulate in the TME and promote immunosuppression [24, 25].

**Fig. 5:**
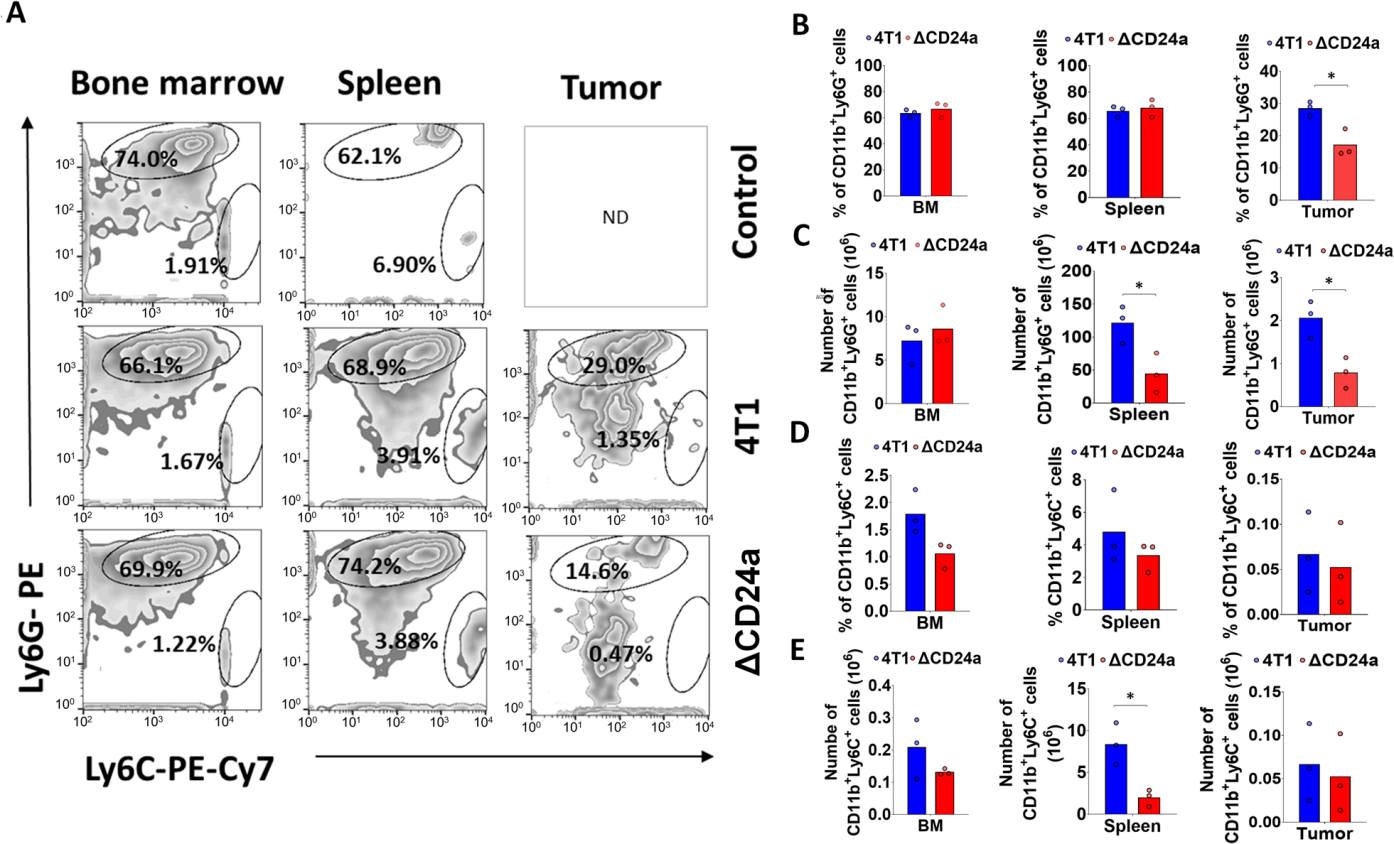
Knocking out CD24a reduces granulocytic and monocytic myeloid-derived suppressor cells (MDSCs) in the spleens and decreases the recruitment of gMDSCs into the TME. **A** Flow cytometry analysis of the distribution of the CD11^+^Ly6G^+^ Ly6C^−^ granulocytic MDSCs (gMDSCs), and CD11b^+^Ly6C^+^Ly6G^−^ monocytic MDSCs (mMDSCs) (n = 3) in the bone marrows, spleens, and tumors from 4T1 tumor-bearing mice and ΔCD24a 4T1 tumor-bearing mice (n = 3). Cells were prepared using the protocol described in the Method section. The isolated cells were stained with FITC-conjugated anti-CD11b, PE-Cy7-conjugated Ly6C^+^, and PE-conjugated Ly6G^+^ antibodies. CD11b^+^ cells were first gated, followed by dot plot analysis using Flow Jo software to identify CD11b^+^Ly6C^+^Ly6G^−^ and CD11^+^Ly6G^+^Ly6C^−^ double-positive populations, respectively. **B** Histograms show mean percentage of gMDSCs population in the tumors, spleens, and bone marrows from ΔCD24a 4T1 tumor-bearing (n = 3) and 4T1 tumor-bearing mice (n = 3). **C** Histograms displayed the cell number of gMDSCs in the tumors, spleens, and bone marrows resected from tumor-bearing mice. **D** Histograms show mean percentage of mMDSCs population in the tumors, spleens, and bone marrows from ΔCD24a 4T1 tumor-bearing (n = 3) and 4T1 tumor-bearing mice (n = 3). **E** Histograms display the cell number of mMDSCs in tumors, spleens, and bone marrows resected from tumor-bearing mice

Figure 5A showed representative flow cytometry analyses of CD11b^+^Ly6G^+^Ly6C^-^ gMDSCs and CD11b^+^Ly6C^+^ Ly6G^-^ mMDSCs in tumors, spleens and bone marrows from ΔCD24a and 4T1 tumor-bearing mice. The results showed a 40% reduction in gMDSCs percentages in the tumors of ΔCD24a tumor-bearing mice as compared to that of the 4T1 tumor-bearing mice, while the percentages of gMDSCs in the spleens and bone marrows were comparable between two groups (Fig. 5A, B). Furthermore, ΔCD24a 4T1 tumor-bearing mice showed a decreased number of gMDSCs in both tumors and spleens, with no significant difference observed in the bone marrows (Fig. 5C). For mMDSCs, ΔCD24a 4T1 tumor-bearing mice showed similar abundance of mMDSCs in spleens and tumors but lower, though statistically insignificant, abundance in bone marrows as compared to those in the 4T1 tumor-bearing mice (Fig. 5D). Moreover, ΔCD24a 4T1 tumor-bearing mice showed a decreased number of mMDSCs in spleens, with similar numbers of mMDSCs in tumors and bone marrows as compared to those in the 4T1 tumor-bearing mice (Fig. 5E). Collectively, these findings indicate that ΔCD24a 4T1 tumors exhibited reduced gMDSC accumulation, likely due to impaired recruitment of gMDSCs from bone marrow to the primary tumor.

### IF staining confirmed that CD24a knockout transformed 4T1 tumors from an immune-suppressive to an immune-active state

**Fig. 6:**
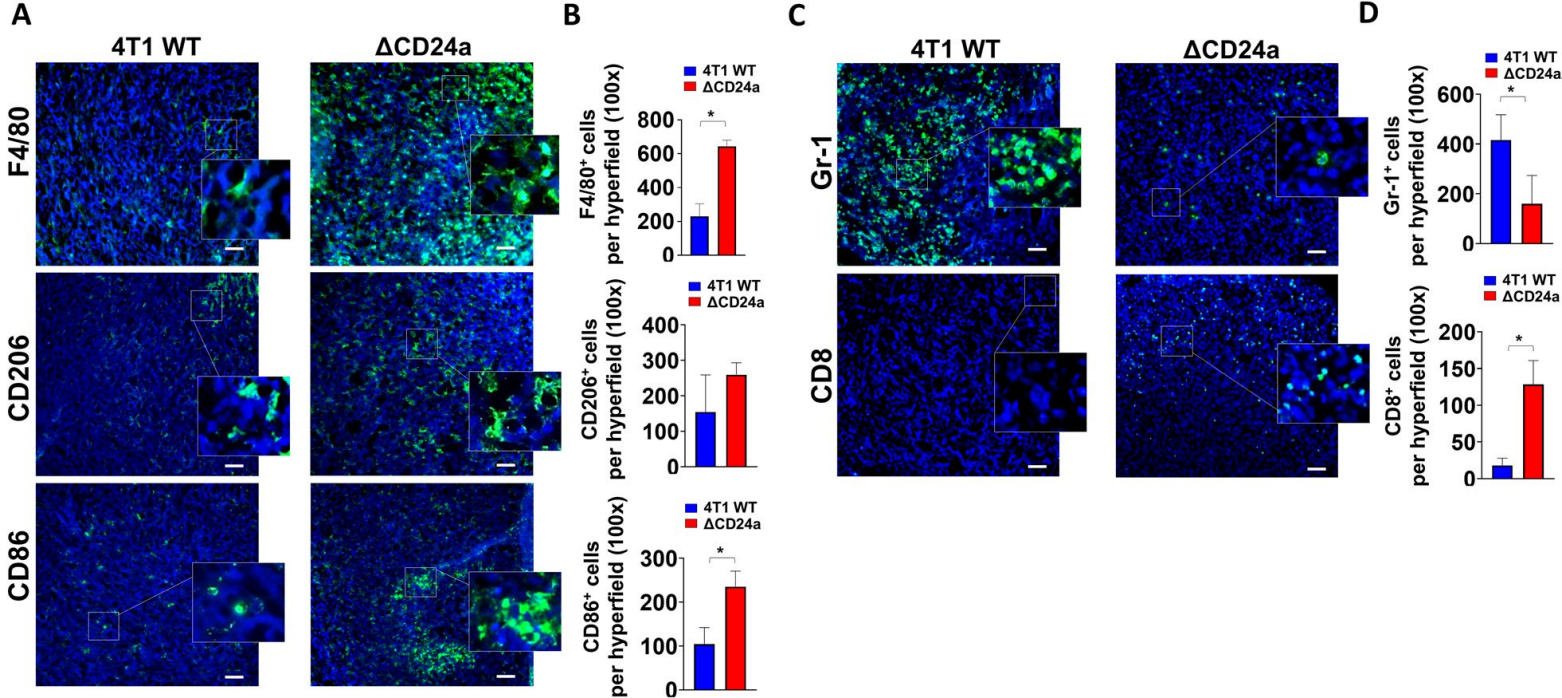
IF staining reveals increased CD86^+^ M1 macrophages and CD8^+^ T cells infiltration in the TME of ΔCD24a 4T1 tumors. **A** Representative immunofluorescence (IF) images of tumor sections showing F4/80⁺, CD206⁺, and CD86⁺ macrophages. The enlarged box highlights the positively stained cells. Frozen sections of ΔCD24a 4T1 tumors and 4T1 tumors were prepared from OCT-embedded frozen tumor tissues for IF staining. Scale bar, 50 μm. **B** Quantitative analysis of IF images for F4/80^+^ macrophages, CD206^+^ macrophages, and CD86^+^ macrophages in tumor sections. P < 0.05. **C** The representative tumor section IF images of Gr-1^+^ MDSCs and CD8^+^ T cells. Scale bar, 50 μm. **D** Quantitative analysis of IF images for Gr-1^+^ MDSCs and CD8.^+^ T cells in tumor sections. P < 0.05

Next, we performed IF staining to examine the distribution of tumor-infiltrating macrophages, MDSCs, and cytotoxic CD8^+^ T cells. The results of IF staining revealed a significant increase in the infiltration of F4/80^+^ macrophages into the TME of ΔCD24a 4T1 compared to 4T1 tumors (Fig. 6A, B). Additionally, the infiltration of CD86^+^ M1 macrophages was substantially higher than CD206^+^ M2 macrophages (Fig. 6A, B). The ΔCD24a 4T1 tumors exhibited a substantial reduction of Gr-1^+^ MDSCs infiltration and a significant increase of cytotoxic CD8^+^ T infiltration as compared to the 4T1 tumors (Fig. 6C, D). Taken together, the IF results confirmed that ΔCD24a 4T1 tumors exhibited an immune active phenotype, characterized by more abundant of tumor-suppressive immune cells including CD86^+^ M1 macrophages and cytotoxic CD8^+^ T cells compared to the pro-tumor immune suppressive phenotype of the 4T1 tumors.

### 3D reconstruction of the TME revealed the distinct spatial immune landscape in ΔCD24a 4T1 versus the WT 4T1 tumors

**Fig. 7:**
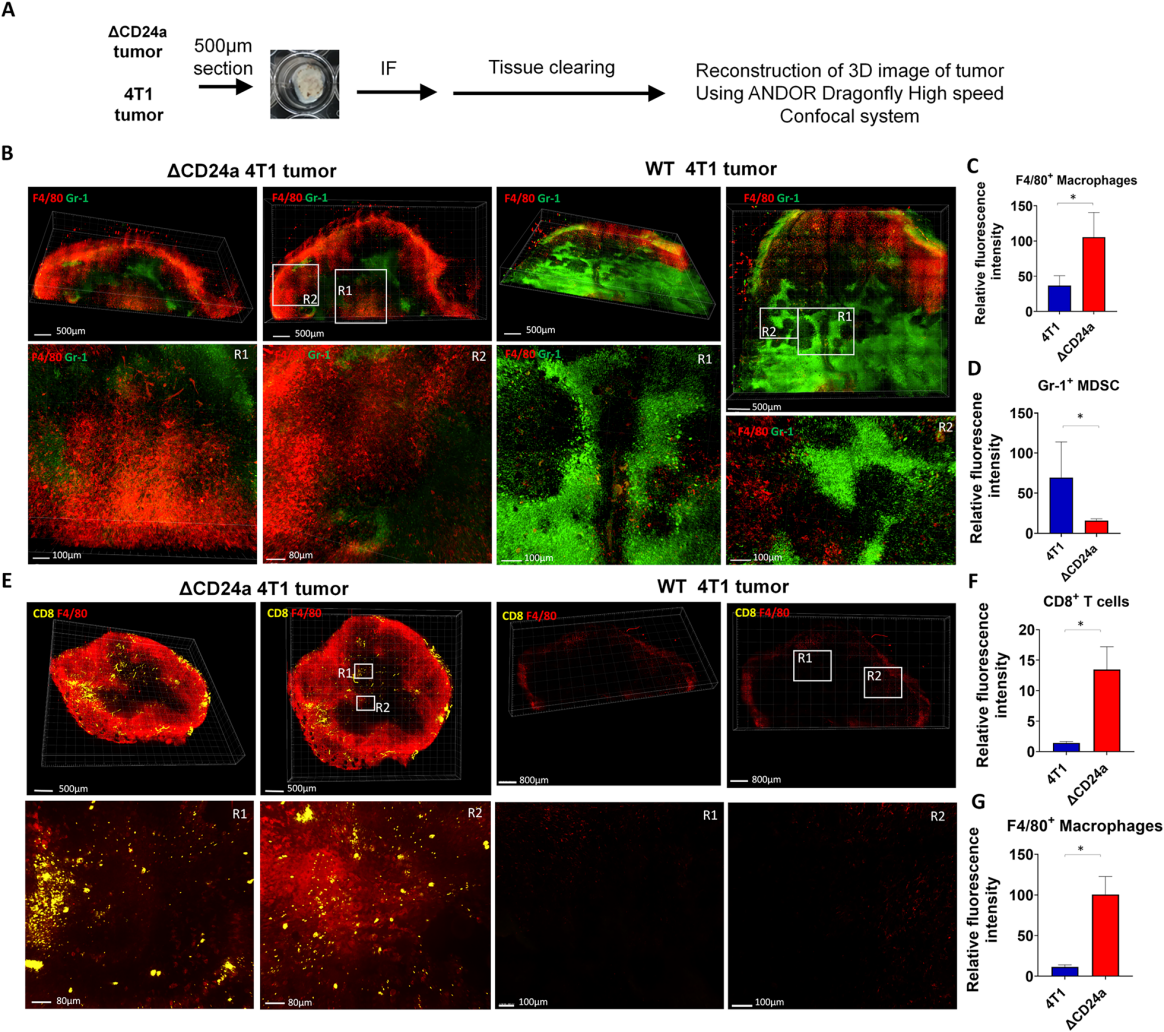
Reconstruction of three-dimensional images confirms macrophages and CD8^+^ T cells infiltration in the TME of ΔCD24a 4T1 tumors. **A** Flow chart of the reconstruction of 3D images of tumors. **B** Fluorescence images of F4/80^+^ macrophages and Gr-1^+^ MDSCs within 4T1 and ΔCD24a 4T1 tumors. **C**, **D** Quantitative fluorescence signals of F4/80^+^ macrophages and Gr-1^+^ MDSCs within 4T1 and ΔCD24a 4T1 tumors. *P < 0.05. **E** Fluorescence images of F4/80^+^ macrophages and CD8^+^ T cells within 4T1 and ΔCD24a 4T1 tumors. **F**, **G** Quantitative fluorescence signals of F4/80^+^ macrophages and CD8^+^ T cells within 4T1 and ΔCD24a 4T1 tumors. Bottom panels of **B** and **E** are enlarged images of the respective frame boxes. *P < 0.05

Subsequently, we employed the IF approach and combined it with a tissue clearance technique, utilizing a high-speed confocal microscopy imaging system to create 3D maps of immune cells in both ΔCD24a 4T1 and 4T1 tumors to further explore the spatial distribution and interactions of the tumor-infiltrating immune cells within the TME (Fig. 7A). The distribution of Gr-1^+^ MDSCs, F4/80^+^ macrophages, and CD8^+^ T cells within the TME was assessed. The results of 3D mapping of TME immune landscape revealed that 4T1 tumors exhibited robust infiltration by Gr-1^+^ MDSCs but had very few F4/80^+^ macrophages (Fig. 7B–D). In contrast, ΔCD24a 4T1 tumors displayed significantly more infiltration of F4/80^+^ macrophages and less of Gr-1^+^ MDSCs (Fig. 6B–D). From the spatial distribution of immune cells, macrophages appeared to be more abundantly distributed at the periphery of the tumor. In contrast, MDSCs are more abundant inside the tumor. Moreover, we observed an increased number of tumor-infiltrating CD8^+^ T cells in the ΔCD24a 4T1 tumors (Fig. 7E–G). CD8^+^ T cells appear to be all over the tumor and appear as punctate spots. These findings further confirm the critical role of CD24a in shaping the immune landscape in the TME.

### CD24a knockout altered the expressions of clinically relevant chemokines in 4T1 cells

**Fig. 8:**
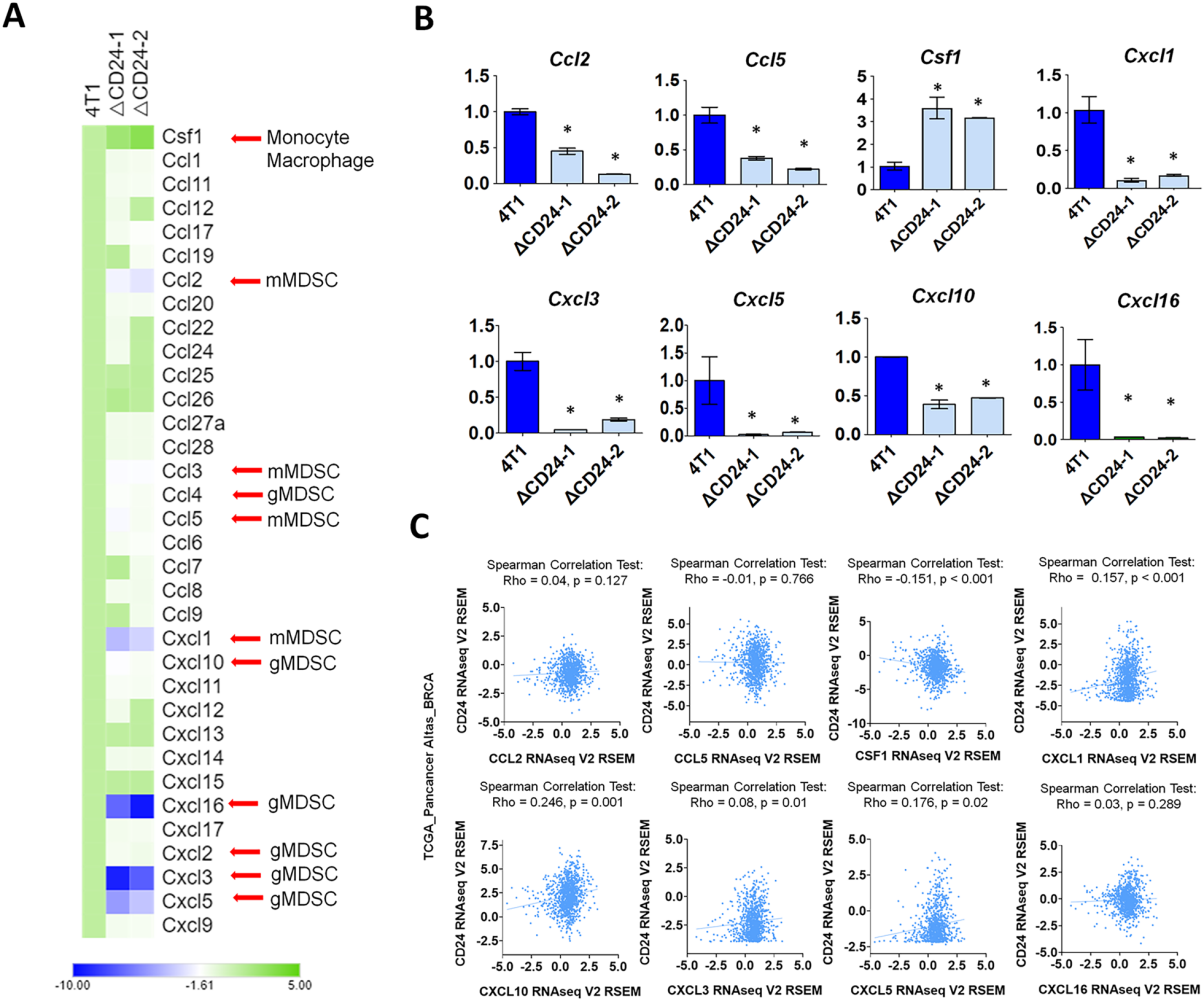
The knockout of CD24a modulates the expression of chemokines crucial for the recruitment of macrophages, and mMDSCs/gMDSCs into the TME. **A** Exon array analysis revealed Chemokine expression profiles between 4T1, ΔCD24-1, and ΔCD24-2 cells. RNA was extracted from 4T1 and ΔCD24a 4T1 cells. The transcriptome analysis was conducted using the Affymetrix GeneChip Human Exon 1.0 ST Array. A heatmap was generated using Transcriptome Analysis Console (TAC) Software. **B** qRT-PCR analysis of chemokine expressions relevant to the recruitment of macrophages and MDSCs. The expression levels of the chemokine genes *Ccl2, Ccl5, Csf1, Cxcl1, Cxcl3, Cxcl5, Cxcl10*, and *Cxcl16* were shown. *P < 0.05. **C** Spearman correlation analysis of chemokines and CD24 expression in a clinical breast cancer database

Previous studies have shown that cancer cell-secreted chemokines play a crucial role in shaping the immunosuppressive TME during tumor progression [25–28]. Therefore, we compared the impact of CD24a knockout on the gene expression patterns between 4T1 cells and two ΔCD24a clones with the focus on the potential alterations of chemokine expressions (Fig. 8A). RT-PCR analysis confirmed that chemokines mediating gMDSCs migration to the TME including CXCL1, CXCL3, CXCL5 and CXCL16, were significantly decreased in ΔCD24a cells (Fig. 8B). Additionally, chemokines such as CCL2, CCL5, CXCL10 are known to attract mMDSC and they were also downregulated in ΔCD24a cells despite that CD24a knockout only had a minor effect on mMDSC recruitment (Figs. 5C, D and 8B). Notably, knockout of CD24a led to a significant increase in M-CSF expression (Fig. 8B). To explore the clinical relevance of our findings, we conducted Spearman correlation analysis of the expression of CD24 and relevant chemokines in TCGA (The Cancer Genome Atlas) Breast Invasive Carcinoma dataset using cBioportal user interface. The results showed that CD24 expression was positively correlated with chemokines involved in the recruitment of gMDSCs, including CXCL1, CXCL3, and CXCL5 in BC patients. Additionally, CD24 expression also correlated with CXCL10 (Fig. 8C). Notably, CD24 expression was inversely correlated with M-CSF expression in BC patients (Fig. 8C). Moreover, re‐expression of CD24a in ΔCD24a cells restored expression of gMDSC-recruiting chemokines CXCL1, CXCL3, and CXCL5 and suppressed M-CSF levels as compared to ΔCD24a cells (Additional file 8: Fig. S7A). In addition, co-culture experiments revealed that the presence of gMDSC-recruiting chemokines, such as CXCL1 and CXCL5, did not alter CD8^+^ T cell-mediated cytotoxicity when 4T1 or ΔCD24a 4T1 cells were co-cultured with CD8^+^ T cells. Similarly, co-culture of 4T1 or ΔCD24a 4T1 cells with macrophages in the presence of these chemokines had no impact on macrophage-mediated phagocytosis (Additional file 8: Fig. S7B). These results suggested that while CD24a might influence the expression of chemokines associated with gMDSC recruitment, the observed immunomodulatory effects appeared to be more directly linked to changes in tumor cell susceptibility to immune effector mechanisms. Indeed, our in vitro co-culture experiments demonstrated that the enhanced sensitivity of ΔCD24a 4T1 cells toward macrophage- and CD8⁺ T cell-mediated cytotoxicity might play a more critical role in driving anti-tumor responses than the indirect effects mediated by altered chemokine expression.

## Discussion

In this study, we investigated the impact of CD24a loss on cancer progression and the immune landscape of the TME using the mouse breast carcinoma cell line 4T1, a murine model of TNBC characterized by poor immunogenicity [29]. We showed that ΔCD24a 4T1 cells exhibited heightened vulnerability to M1 macrophage-mediated phagocytosis and CD8^+^ T cell-mediated cytotoxicity (Fig. 1B, C). In vivo depletion of macrophages or CD8^+^ T cells abrogated the tumor growth suppression observed in ΔCD24a 4T1 tumor-bearing mice (Fig. 1F, G), underscoring the essential roles of these immune effector cells in mediating CD24a loss-associated anti-tumor effects. Furthermore, macrophage depletion only promoted tumor growth in the ΔCD24a model while had no effect on 4T1 model (Fig. 1D–G), suggesting a pivotal role of CD24a in macrophage-driven anti-tumor immunity. Knocking out CD24a in 4T1 tumors was associated with delayed tumor growth (Fig. 2). Notably, CD24a knockout substantially increased intratumoral infiltration of cytotoxic CD8^+^ T cells and M1 macrophages, thereby transitioning the TME from an immunosuppressive ('cold') to an immunogenic ('hot') state (Figs. 6 and 7). In addition, ΔCD24a 4T1 cells exhibited reduced expression of chemokines linked to gMDSC recruitment (Fig. 8), which may represent an indirect or secondary contribution to the observed enhancement of anti-tumor immunity.

The immune landscape of tumors is defined by many crucial factors including immune contexture of the TME and cancer cells themselves [30]. TNBC often develops resistance to first-line chemotherapy, and accumulates mutations, leading to the transformation of the TME into a “cold” state [31]. This transformation is marked by decreased CD8^+^ T cell infiltration, rendering the tumors less responsive to immune checkpoint inhibitor (ICI) therapy [32]. The molecular heterogeneity of TNBC such as the accumulation of tumor mutation burdens and the activation of pathways like NF-κB, PTEN/PI3K/AKT/mTOR, JAK/STAT, and RTKs, contributes to chemoresistance and tumor progression [33]. However, targeted therapeutics for these pathways have proven ineffective for TNBC patients [33]. CD24 has recently been identified as a central molecule in tumor immune evasion by interacting with Siglec-10 on macrophages in human, sending a “don’t eat me” signal that prevents phagocytosis [19]. However, whether CD24 directly contributes to fostering an immune-suppressive TME is poorly investigated. In this study, we demonstrated that knocking out the murine *Cd24a* gene in 4T1 cells significantly increased the sensitivity to macrophages (Fig. 1C). *In vivo* depletion studies further confirmed the critical role of macrophages in mediating the anti-tumor effects of CD24a loss. Depletion of macrophages significantly reversed tumor growth inhibition observed in ΔCD24a tumors (Fig. 1F, G), highlighting the essential role of macrophages in orchestrating immune-mediated tumor inhibition. In addition, ΔCD24a tumors displayed an elevated infiltration of macrophages in the TME (Fig. 3A–C). Moreover, Exon array and RT-PCR analyses revealed that the knockout of CD24a resulted in a substantial increase in M-CSF expression (Fig. 8A, B), suggesting that the presence of CD24a not only transmits a “don’t eat me” signal to inhibit phagocytosis but also modulates M-CSF expression to inhibit the infiltration and maturation of macrophages within the TME. Notably, we established a clinical link between CD24 and M-CSF expression in breast cancer patients, demonstrating that CD24 expression negatively correlates with M-CSF expression in breast cancer patients (Fig. 8C). Furthermore, our IF results indicated that a substantial increase of CD86^+^ M1 macrophages but not CD206^+^ M2 macrophages was observed in ΔCD24a tumors as compared to 4T1 tumors (Fig. 6A).

Our results showed a 40% reduction in gMDSCs percentages in the tumors of ΔCD24a tumor-bearing mice as compared to the 4T1 tumor-bearing mice, while the percentages of gMDSCs in the spleens and bone marrows were comparable between two groups (Fig. 5A, B). Furthermore, ΔCD24a 4T1 tumor-bearing mice showed a decreased number of gMDSCs in both tumors and spleens, with no significant difference observed in the bone marrows (Fig. 5C). For mMDSCs, ΔCD24a 4T1 tumor-bearing mice showed similar percentages of mMDSCs in spleens and tumors but lower, though statistically insignificant, percentages in bone marrows as compared to the 4T1 tumor-bearing mice (Fig. 5D). Moreover, ΔCD24a 4T1 tumor-bearing mice showed a decreased number of mMDSCs in spleens, and with similar numbers of mMDSCs in tumors and bone marrows as compared to the 4T1 tumor-bearing mice (Fig. 5E). Collectively, these findings indicate that ΔCD24a 4T1 tumors exhibited reduced gMDSC accumulation, likely attributable to impaired recruitment of gMDSCs from bone marrow to the primary tumor. Previous studies have shown that both gMDSCs and mMDSCs play significant roles in promoting tumor progression by suppressing cytotoxic T cell activation and proliferation via different mechanisms [34–39]. Additionally, we showed that knockout of CD24a led to a significant increase in CD8^+^ T cell infiltration within ΔCD24a 4T1 tumors as compared to 4T1 tumors (Fig. 3). This observation may be attributed to a reduction in gMDSCs, which are known to play a crucial role in suppressing the recruitment and infiltration of CD8^+^ T cell into TME [22, 25]. The gMDSCs primarily produce reactive oxygen species (ROS) and nitric oxide (NO) to suppress T cell function [38]. On the other hand, mMDSCs produce immunosuppressive cytokines like TGF-beta and IL-10 and have higher levels of arginase activity, which has been shown to compete arginine as the energy resource with TILs [24, 39]. We showed that the number of gMDSCs in the TME was approximately thirty times greater than mMDSCs, indicating that gMDSCs play a more significant role in immunosuppression during tumor progression than mMDSCs (Fig. 5C, E). Our results showed that CD24a knockout negatively regulated the expression of chemokines involved in the recruitment of gMDSCs, including, CXCL1, CXCL3, and CXCL5, and CXCL16 (Fig. 8A, B) [40, 41], leading to a decreased gMDSC infiltration to the TME (Fig. 5A–C). Importantly, our findings extended beyond the syngeneic mouse model; clinically, we demonstrated a significant correlation between the expression of CD24 and CXCL1, CXCL3, and CXCL5 (Fig. 8C). Additionally, we found that CD24a knockout also had a minor effect on suppressing mMDSCs recruitment to the TME despite the low number of mMDSC infiltrates in the TME. This finding may be explained by a decreased expression of several mMDSC-related chemokines such as CCL2, CCL5, and CXCL10 in ΔCD24a cells (Fig. 8A, B) [42, 43].

In our previous study, we demonstrated that CD24 knockdown reduced the activation of key RTKs, including EGFR and MET [13]. This observation is significant given that certain RTKs have been implicated in the upregulation of chemokines, contributing to immune modulation and tumor progression [44]. In the present study, we similarly found that CD24a knockout in the 4T1 model reduced EGFR protein expression and attenuated EGF-induced EGFR phosphorylation. These results suggest a potential mechanistic link between CD24a loss and the downregulation of cytokines and chemokines observed in CD24a-deficient tumors (Fig. 8). In addition to altering the immune landscape, reduced EGFR activity may also decrease the intrinsic tumorigenic capacity of 4T1 cells. Our in vivo depletion experiments targeting macrophages and CD8⁺ T cells revealed that ΔCD24a tumor-bearing mice exhibited delayed tumor growth during the first three weeks following macrophage depletion (Fig. 1F); however, tumor growth subsequently accelerated, ultimately resulting in significantly larger tumors compared to the control antibody-treated mice (Fig. 1F). A similar pattern was observed in ΔCD24a tumor-bearing mice following CD8⁺ T cell depletion (Fig. 1F). These findings suggested that, in addition to immune-mediated effects, CD24a deficiency may also exert tumor-intrinsic growth-suppressive effects, potentially accounting for the observed delay in tumor progression. This was supported by our in vitro data demonstrating that CD24a loss impaired EGFR signaling and reduced the sphere-forming capacity of 4T1 cells. Taken together, both the intrinsic tumor growth delay of 4T1 cells and enhanced anti-tumor immune responses associated with CD24a depletion likely contribute to the observed suppression of tumor growth and possibly also metastasis.

In-depth examination of the TME using 3D reconstruction techniques enabled us to compare spatial immune cell infiltration patterns between 4T1 tumors and ΔCD24a 4T1 tumors. By reconstructing tumor sections with up to 500 μm thickness, this method provides a comprehensive 3D perspective on the spatial distribution and interactions of tumor-infiltrating immune cells. The 3D reconstruction confirmed that 4T1 tumors had substantial infiltration by Gr-1^+^ MDSCs but very few F4/80^+^ macrophages. In contrast, ΔCD24a 4T1 tumors displayed significant infiltration by F4/80^+^ macrophages instead of Gr-1^+^ MDSCs (Fig. 7B–D). Additionally, ΔCD24a 4T1 tumors showed an increased number of tumor-infiltrating CD8^+^ T cells (Fig. 7E–G). Furthermore, the effects of CD24a knockout on tumor growth and anti-tumor immune response were confirmed in a Matrigel-free orthotopic 4T1 BALB/c model (Additional file 9: Fig. S8A and S8B), indicating that the observed results are independent of Matrigel. These findings demonstrate that CD24a plays a crucial role in modulating the TME. Specifically, the absence of CD24a shifts an immune-suppressive TME dominated by gMDSCs to an immune-active TME with increased macrophage and CD8^+^ T cell infiltration, effectively transforming the TME from "cold" to "hot" anti-tumor immune state. This transformation is significant as it suggests that targeting CD24 could enhance anti-tumor immune responses and improve the efficacy of immunotherapies.

In addition, previous studies have shown that the lack of CD24 in human breast cancer leads to favor tumor initiation and growth [45, 46]. However, our prior work showed that CD24 is essential for tumor growth and metastatic progression in human TNBC cells, as it promotes oncogenic signaling via EGFR and MET [13]. In our current study, depletion of CD24a expression in murine 4T1 breast cancer cells resulted in reduction of tumor growth and progression. This discrepancy may be explained by the complex molecular subtypes of human breast cancer, which harbors various oncogenic signaling as opposed to the mouse breast cancer [45]. In addition, Cremers, et al. showed that CD24 is not required for tumor initiation and growth in murine breast cancer model of MMTV-PyMT (the mouse mammary tumor virus-the polyoma virus middle T antigen). This model, driven by mammary gland-specific expression of the polyoma virus middle T antigen, generates tumors with distinct genetic and molecular characteristics compared to the 4T1 TNBC model. Consequently, the oncogenic role of CD24a appears to be less prominent in PyMT-driven breast tumors [47]. Moreover, Fico et al. showed that the Lin⁻CD24⁺CD90⁺ population displayed enhanced metastatic capacity compared to Lin⁻ALDH1⁺CD90⁻ cells in MMTV-PyMT model, suggesting that CD24 is dispensable for primary tumor initiation but is crucial for metastasis formation in MMTV-PyMT model [48]. These results align with our previous observations in human TNBC xenograft models, where CD24 expression was significantly upregulated during metastatic lung colonization [13]. Together, these findings underscore the multifaceted and context-dependent role of CD24 in breast cancer progression.

## Conclusions

Our study reveals that CD24a contributes to immune evasion and tumor progression in the murine TNBC model. The loss of CD24a was associated with delayed tumor growth and a shift in the TME toward a more immunologically active state, characterized by enhanced anti-tumor activity of macrophages, and cytotoxic CD8^+^ T cells, along with reduced gMDSCs accumulation. Our findings support the notion that targeting CD24 could be a promising strategy for enhancing anti-tumor activity, particularly in immune “cold” TNBC tumors. Future studies may explore whether CD24-targeted therapies could promote the efficacy of immune checkpoint inhibitors and broaden the therapeutic scope of cancer immunotherapy across CD24-positive solid tumors.

## Supplementary Information

Additional file 1.

Additional file 2.

Additional file 3.

Additional file 4.

Additional file 5.

Additional file 6.

Additional file 7.

Additional file 8.

Additional file 9.

## Data Availability

All data are included in this published article and Additional files.

## Abbreviations

3D: Three dimensional
ALDH1: Aldehyde dehydrogenase 1
ATCC: American type culture collection
BMDM: Bone marrow-derived macrophage
CRISPR: Clustered regularly interspaced short palindromic repeats
DAPI: 4',6-Diamidino-2-Phenylindole
DMEM: Dulbecco’s modified eagle medium
EGFR: Epidermal growth factor receptor
Erk: Extracellular signal-related kinase
FITC: Fluorescein isothiocyanate
ICI: Immune checkpoint inhibitor
IFN-γ: Interferon-Gamma
M-CSF: Macrophage colony-stimulating factor
MDSCs: Myeloid-derived suppressor cells
MET: Met proto-oncogene
NK cell: Natural killer cells
PBS: Phosphate-Buffered saline
PKB: Protein kinase B
qPCR: Quantitative polymerase chain reaction
RBC: Red blood cell
RTK: Receptor tyrosine kinase
TAMs: Tumor-associated macrophages
TCGA: The cancer genome atlas
TME: Tumor microenvironment
TNBC: Triple-negative breast cancer
IF: Immunofluorescence

## References

[1] Kristiansen G, Sammar M, Altevogt P. Tumour biological aspects of CD24, a mucin-like adhesion molecule. J Mol Histol. 2004;35(3):255–62. 15339045 10.1023/b:hijo.0000032357.16261.c5

[2] Fang X, Zheng P, Tang J, Liu Y. CD24: from a to z. Cell Mol Immunol. 2010;7(2):100–3. 20154703 10.1038/cmi.2009.119 PMC4001892

[3] Altevogt P, Sammar M, Hüser L, Kristiansen G. Novel insights into the function of CD24: a driving force in cancer. Int J Cancer. 2021;148(3):546–59. 32790899 10.1002/ijc.33249

[4] Carrion C, Guérin E, Gachard N, le Guyader A, Giraut S, Feuillard J. Adult bone marrow three-dimensional phenotypic landscape of B-cell differentiation. Cytometry B Clin Cytom. 2019;96(1):30–8. 30450798 10.1002/cyto.b.21747

[5] Zhang X, Yu C, Liu JQ, Bai XF. Dendritic cell expression of CD24 contributes to optimal priming of T lymphocytes in lymph nodes. Front Immunol. 2023;14:1116749. 36969215 10.3389/fimmu.2023.1116749 PMC10033833

[6] Chen GY, Tang J, Zheng P, Liu Y. CD24 and Siglec-10 selectively repress tissue damage-induced immune responses. Science. 2009;323(5922):1722–5. 19264983 10.1126/science.1168988 PMC2765686

[7] Eyvazi S, Kazemi B, Dastmalchi S, Bandehpour M. Involvement of CD24 in multiple cancer related pathways makes it an interesting new target for cancer therapy. Curr Cancer Drug Targets. 2018;18(4):328–36. 28820056 10.2174/1570163814666170818125036

[8] Aigner S, Ramos CL, Hafezi-Moghadam A, Lawrence MB, Friederichs J, Altevogt P, et al. CD24 mediates rolling of breast carcinoma cells on P-selectin. FASEB J. 1998;12(12):1241–51. 9737727 10.1096/fasebj.12.12.1241

[9] Friederichs J, Zeller Y, Hafezi-Moghadam A, Gröne H-J, Ley K, Altevogt P. The CD24/P-selectin binding pathway initiates lung arrest of human A125 adenocarcinoma cells. Cancer Res. 2000;60(23):6714–22. 11118057

[10] Baumann P, Cremers N, Kroese F, Orend G, Chiquet-Ehrismann R, Uede T, et al. CD24 expression causes the acquisition of multiple cellular properties associated with tumor growth and metastasis. Cancer Res. 2005;65(23):10783–93. 16322224 10.1158/0008-5472.CAN-05-0619

[11] Baumann P, Thiele W, Cremers N, Muppala S, Krachulec J, Diefenbacher M, et al. CD24 interacts with and promotes the activity of c-src within lipid rafts in breast cancer cells, thereby increasing integrin-dependent adhesion. Cell Mol Life Sci. 2012;69(3):435–48. 21710320 10.1007/s00018-011-0756-9 PMC11114536

[12] Ahmad F, Dina K. CD24 induces the activation of β-catenin in intestinal tumorigenesis. J Cancer Sci Ther. 2016;08(5):135–42.

[13] Chan SH, Tsai KW, Chiu SY, Kuo WH, Chen HY, Jiang SS, et al. Identification of the novel role of CD24 as an oncogenesis regulator and therapeutic target for triple-negative breast cancer. Mol Cancer Ther. 2019;18(1):147–61. 30381446 10.1158/1535-7163.MCT-18-0292

[14] Charafe-Jauffret E, Ginestier C, Bertucci F, Cabaud O, Wicinski J, Finetti P, et al. ALDH1-positive cancer stem cells predict engraftment of primary breast tumors and are governed by a common stem cell program. Cancer Res. 2013;73(24):7290–300. 24142344 10.1158/0008-5472.CAN-12-4704

[15] Li H, Ma F, Wang H, Lin C, Fan Y, Zhang X, et al. Stem cell marker aldehyde dehydrogenase 1 (ALDH1)-expressing cells are enriched in triple-negative breast cancer. Int J Biol Markers. 2013;28(4):e357–64. 24338721 10.5301/jbm.5000048

[16] Li YH, Sun X, Wang HB. Role of CD24 in anoikis resistance of ovarian cancer cells. J Huazhong Univ Sci Technolog Med Sci. 2015;35(3):390–6. 26072079 10.1007/s11596-015-1443-0

[17] Nagare RP, Sneha S, Sidhanth C, Roopa S, Murhekar K, Shirley S, et al. Expression of cancer stem cell markers CD24, EPHA1 and CD9 and their correlation with clinical outcome in epithelial ovarian tumours. Cancer Biomark. 2020;28(3):397–408. 32224528 10.3233/CBM-201463 PMC12662368

[18] Yang X, Sarvestani SK, Moeinzadeh S, He X, Jabbari E. Effect of CD44 binding peptide conjugated to an engineered inert matrix on maintenance of breast cancer stem cells and tumorsphere formation. PLoS ONE. 2013;8(3): e59147. 23527117 10.1371/journal.pone.0059147 PMC3601067

[19] Barkal AA, Brewer RE, Markovic M, Kowarsky M, Barkal SA, Zaro BW, et al. CD24 signalling through macrophage Siglec-10 is a target for cancer immunotherapy. Nature. 2019;572(7769):392–6. 31367043 10.1038/s41586-019-1456-0 PMC6697206

[20] Almeida JL, Korch CT, et al. Authentication of human and mouse cell lines by short tandem repeat (STR) DNA genotype analysis. In: Markossian S, Grossman A, Arkin M, Auld D, Austin C, Baell J, et al., editors. Assay guidance manual. Bethesda: Eli Lilly & Company; 2004. 23805434

[21] Sanger F, Coulson AR, Barrell BG, Smith AJH, Roe BA. Cloning in single-stranded bacteriophage as an aid to rapid DNA sequencing. J Mol Biol. 1980;143(2):161–78. 6260957 10.1016/0022-2836(80)90196-5

[22] Jian S-L, Chen W-W, Su Y-C, Su Y-W, Chuang T-H, Hsu S-C, et al. Glycolysis regulates the expansion of myeloid-derived suppressor cells in tumor-bearing hosts through prevention of ROS-mediated apoptosis. Cell Death Dis. 2017;8(5): e2779. 28492541 10.1038/cddis.2017.192 PMC5520713

[23] Kuo WH, Chu PY, Wang CC, Huang PS, Chan SH. MAP7D3, a novel prognostic marker for triple-negative breast cancer, drives cell invasiveness and cancer-initiating cell properties to promote metastatic progression. Biol Direct. 2023;18(1):44. 37550720 10.1186/s13062-023-00400-x PMC10405500

[24] Groth C, Hu X, Weber R, Fleming V, Altevogt P, Utikal J, et al. Immunosuppression mediated by myeloid-derived suppressor cells (MDSCs) during tumour progression. Br J Cancer. 2019;120(1):16–25. 30413826 10.1038/s41416-018-0333-1 PMC6325125

[25] Lu J, Luo Y, Rao D, Wang T, Lei Z, Chen X, et al. Myeloid-derived suppressor cells in cancer: therapeutic targets to overcome tumor immune evasion. Exp Hematol Oncol. 2024;13(1):39. 38609997 10.1186/s40164-024-00505-7 PMC11010322

[26] Wang X, Liu M, Zhang J, Brown NK, Zhang P, Zhang Y, et al. CD24-Siglec axis is an innate immune checkpoint against metaflammation and metabolic disorder. Cell Metab. 2022;34(8):1088–103. 35921817 10.1016/j.cmet.2022.07.005 PMC9393047

[27] Hao Z, Li R, Wang Y, Li S, Hong Z, Han Z. Landscape of myeloid-derived suppressor cell in tumor immunotherapy. Biomark Res. 2021;9(1):77. 34689842 10.1186/s40364-021-00333-5 PMC8543853

[28] Ozbay Kurt FG, Lasser S, Arkhypov I, Utikal J, Umansky V. Enhancing immunotherapy response in melanoma: myeloid-derived suppressor cells as a therapeutic target. J Clin Invest. 2023. 10.1172/JCI170762. 37395271 10.1172/JCI170762 PMC10313369

[29] Wu M, Zheng D, Zhang D, Yu P, Peng L, Chen F, et al. Converting immune cold into hot by biosynthetic functional vesicles to boost systematic antitumor immunity. iScience. 2020;23(7): 101341. 32683314 10.1016/j.isci.2020.101341 PMC7371908

[30] Galon J, Bruni D. Approaches to treat immune hot, altered and cold tumours with combination immunotherapies. Nat Rev Drug Discov. 2019;18(3):197–218. 30610226 10.1038/s41573-018-0007-y

[31] Wu B, Zhang B, Li B, Wu H, Jiang M. Cold and hot tumors: from molecular mechanisms to targeted therapy. Signal Transduct Target Ther. 2024;9(1):274. 39420203 10.1038/s41392-024-01979-x PMC11491057

[32] Geurts V, Kok M. Immunotherapy for metastatic triple negative breast cancer: current paradigm and future approaches. Curr Treat Options Oncol. 2023;24(6):628–43. 37079257 10.1007/s11864-023-01069-0 PMC10172210

[33] Kumari M, Krishnamurthy PT, Sola P. Targeted drug therapy to overcome chemoresistance in triple-negative breast cancer. Curr Cancer Drug Targets. 2020;20(8):559–72. 32370716 10.2174/1568009620666200506110850

[34] Srivastava MK, Sinha P, Clements VK, Rodriguez P, Ostrand-Rosenberg S. Myeloid-derived suppressor cells inhibit T-cell activation by depleting cystine and cysteine. Cancer Res. 2010;70(1):68–77. 20028852 10.1158/0008-5472.CAN-09-2587 PMC2805057

[35] Feng S, Cheng X, Zhang L, Lu X, Chaudhary S, Teng R, et al. Myeloid-derived suppressor cells inhibit T cell activation through nitrating LCK in mouse cancers. Proc Natl Acad Sci USA. 2018;115(40):10094–9. 30232256 10.1073/pnas.1800695115 PMC6176562

[36] Pinton L, Solito S, Damuzzo V, Francescato S, Pozzuoli A, Berizzi A, et al. Activated T cells sustain myeloid-derived suppressor cell-mediated immune suppression. Oncotarget. 2016;7(2):1168–84. 26700461 10.18632/oncotarget.6662 PMC4811451

[37] Monu NR, Frey AB. Myeloid-derived suppressor cells and anti-tumor T cells: a complex relationship. Immunol Invest. 2012;41(6–7):595–613. 23017137 10.3109/08820139.2012.673191 PMC3701882

[38] Lu T, Gabrilovich DI. Molecular pathways: tumor-infiltrating myeloid cells and reactive oxygen species in regulation of tumor microenvironment. Clin Cancer Res. 2012;18(18):4877–82. 22718858 10.1158/1078-0432.CCR-11-2939 PMC3445728

[39] Raber P, Ochoa AC, Rodríguez PC. Metabolism of L-arginine by myeloid-derived suppressor cells in cancer: mechanisms of T cell suppression and therapeutic perspectives. Immunol Invest. 2012;41(6–7):614–34. 23017138 10.3109/08820139.2012.680634 PMC3519282

[40] Cheng R, Billet S, Liu C, Haldar S, Choudhury D, Tripathi M, et al. Periodontal inflammation recruits distant metastatic breast cancer cells by increasing myeloid-derived suppressor cells. Oncogene. 2020;39(7):1543–56. 31685946 10.1038/s41388-019-1084-z PMC7018659

[41] Ouzounova M, Lee E, Piranlioglu R, El Andaloussi A, Kolhe R, Demirci MF, et al. Monocytic and granulocytic myeloid derived suppressor cells differentially regulate spatiotemporal tumour plasticity during metastatic cascade. Nat Commun. 2017;8:14979. 28382931 10.1038/ncomms14979 PMC5384228

[42] Kumar V, Patel S, Tcyganov E, Gabrilovich DI. The nature of myeloid-derived suppressor cells in the tumor microenvironment. Trends Immunol. 2016;37(3):208–20. 26858199 10.1016/j.it.2016.01.004 PMC4775398

[43] Sun Y, Mo Y, Jiang S, Shang C, Feng Y, Zeng X. CXC chemokine ligand-10 promotes the accumulation of monocyte-like myeloid-derived suppressor cells by activating p38 MAPK signaling under tumor conditions. Cancer Sci. 2023;114(1):142–51. 36168841 10.1111/cas.15598 PMC9807505

[44] Wu YM, Robinson DR, Kung HJ. Signal pathways in up-regulation of chemokines by tyrosine kinase MER/NYK in prostate cancer cells. Cancer Res. 2004;64(20):7311–20. 15492251 10.1158/0008-5472.CAN-04-0972

[45] Fillmore C, Kuperwasser C. Human breast cancer stem cell markers CD44 and CD24: enriching for cells with functional properties in mice or in man? Breast Cancer Res. 2007;9(3):303. 17540049 10.1186/bcr1673 PMC1929090

[46] Stingl J, Eirew P, Ricketson I, Shackleton M, Vaillant F, Choi D, et al. Purification and unique properties of mammary epithelial stem cells. Nature. 2006;439(7079):993–7. 16395311 10.1038/nature04496

[47] Herschkowitz JI, Simin K, Weigman VJ, Mikaelian I, Usary J, Hu Z, et al. Identification of conserved gene expression features between murine mammary carcinoma models and human breast tumors. Genome Biol. 2007;8(5):R76. 17493263 10.1186/gb-2007-8-5-r76 PMC1929138

[48] Fico F, Bousquenaud M, Rüegg C, Santamaria-Martínez A. Breast cancer stem cells with tumor-versus metastasis-initiating capacities are modulated by TGFBR1 inhibition. Stem Cell Reports. 2019;13(1):1–9. 31257133 10.1016/j.stemcr.2019.05.026 PMC6626885

