# Supplementary material for: CD24a knockout results in an enhanced macrophage- and CD8⁺ T cell-mediated anti-tumor immune responses in tumor microenvironment in a murine triple-negative breast cancer model
Source: J Biomed Sci. 2025 Aug 9;32:73. doi: 10.1186/s12929-025-01165-3 (PMC12335121; doi:10.1186/s12929-025-01165-3)
Supplement: Supplementary file 1 — Additional file 1. [file 12929_2025_1165_MOESM1_ESM.docx]

**Supplementary Table S1. Antibodies, reagents, and chemicals.**

| Items | Cat. number | Manufacturer |
| --- | --- | --- |
| FITC-conjugated anti-CD11b antibody | 101206 | Biolegend, San Diego, CA, USA |
| PE-Cy7-conjugated anti-Ly6C antibody | 128018 | Biolegend, San Diego, CA, USA |
| PE-conjugated anti-Ly6G antibody | 164504 | Biolegend, San Diego, CA, USA |
| PerCP/Cy5.5-conjugated anti-F4/80 antibody | 123128 | Biolegend, San Diego, CA, USA |
| FITC-conjugated anti-CD49b^+^ antibody | 108905 | Biolegend, San Diego, CA, USA |
| FITC anti-mouse CD3 Antibody | 100203 | Biolegend, San Diego, CA, USA |
| PerCP/Cyanine5.5 anti-mouse CD8a Antibody | 155014 | Biolegend, San Diego, CA, USA |
| Accutase | 423201 | Biolegend, San Diego, CA, USA |
| PE anti-mouse CD24 Antibody | 138504 | Biolegend, San Diego, CA, USA |
| PE Rat IgG2c, κ Isotype Ctrl Antibody | 400707 | Biolegend, San Diego, CA, USA |
| FITC Mouse IgG2b, κ Isotype Ctrl Antibody | 402208 | Biolegend, San Diego, CA, USA |
| PE/Cyanine7 Rat IgG2c, κ Isotype Ctrl Antibody | 400721 | Biolegend, San Diego, CA, USA |
| PerCP/Cyanine5.5 Mouse IgG2a, κ Isotype Ctrl Antibody | 400258 | Biolegend, San Diego, CA, USA |
| Ficoll-Paque™ PLUS | 17144003 | Cytiva, Logan, UT, USA |
| Anti-F4/80 antibody | 30325 | Cell signaling Technology, Denver, MA, USA |
| Anti-CD86 antibody | 91882 | Cell signaling Technology, Denver, MA, USA |
| Anti-CD206 antibody | 24595 | Cell signaling Technology, Denver, MA, USA |
| Anti-Gr-1 antibody | 31469 | Cell signaling Technology, Denver, MA, USA |
| Anti- CD8α antibody | ab33786 | Abcam, Cambridge, CB2 0AX, UK |
| 0.25% Trypsin-EDTA | 25200056 | ThermoFisher Scientific Inc., Waltham, MA, USA |
| anti-EGFR antibody | #4267 | Cell Signaling Technology, Danvers, MA, USA |
| anti-phospho-EGFR antibody | #3777 | Cell Signaling Technology, Danvers, MA, USA |
| anti-GAPDH antibody | #5174 | Cell Signaling Technology, Danvers, MA, USA |
| Anti-rabbit IgG, HRP-linked Antibody | #7074 | Cell Signaling Technology, Danvers, MA, USA |
| Protease and Phosphatase Inhibitor Cocktail | PPC1010 | Sigma-Aldrich, St. Louis, MO, USA |
| RapiClear CS solution | RCCS001 | SunJin Lab Co., Hsinchu, Taiwan |
| RNase A | 10109142001 | Sigma-Aldrich, St. Louis, MO, USA |
| propidium iodide solution | P4864-10ML | Sigma-Aldrich, St. Louis, MO, USA |
| Anti-mouse CSF1R | BE0213 | Bio X Cell, Hanover, NH, USA |
| Rat IgG2a isotype control | BE0089 | Bio X Cell, Hanover, NH, USA |
| Anti-mouse CD8α | mcd8-mab10-1 | InvivoGen, San Diego, CA , USA |
| EasySep™ Mouse NK Cell Isolation Kit | 19855 | Stemcell technologies, Vancouver, Canada |
| EasySep™ Mouse CD8+ T Cell Isolation Kit | 19853 | Stemcell technologies, Vancouver, Canada |
| Mouse Recombinant IFN-gamma | 78021 | Stemcell technologies, Vancouver, Canada |
| Mouse Recombinant M-CSF | 78059 | Stemcell technologies, Vancouver, Canada |

**Supplementary Table S2. Sequences of qRT-PCR primers**

| Gene | Sequences | Manufacturer |
| --- | --- | --- |
| *Ccl2*  NM_031530 | Forward: AGCCAACTCTCACTGAAGCC  Reverse: AACTGTGAACAACAGGCCCA | MDBio, Inc., New Taipei City, Taiwan |
| *Ccl5*  NM_013653 | Forward: GTGCCAACCCAGAGAAGAA  Reverse: GGAAGCTATACAGGGTCAGAATC | MDBio, Inc., New Taipei City, Taiwan |
| *Csf1*  NM_007778 | Forward: CTTTCCATCCTCACCCTTAGAC  Reverse: CTCCAGGGCCCACAATAAATA | MDBio, Inc., New Taipei City, Taiwan |
| *Cxcl3*  NM_203320 | Forward: CATGCCGGTGTAGGAAGAAT  Reverse: TTCATGTGACACCGTAAGACC | MDBio, Inc., New Taipei City, Taiwan |
| *Cxcl5* NM_009141 | Forward: TGCCTGAAGGAAGAGAGAGA  Reverse: TGGAGGAGGTGTGGAGATT | MDBio, Inc., New Taipei City, Taiwan |
| *Cxcl10*  NM_021274 | Forward: TCCTAATTGCCCTTGGTCTTC  Reverse: CATGGCTTGACCATCATCCT | MDBio, Inc., New Taipei City, Taiwan |
| *Cxcl16*  NM_001017478 | Forward: CATCTGCCCTCGTCTACTTTC  Reverse: GAGGTTGGCTTGGGCTAAATA | MDBio, Inc., New Taipei City, Taiwan |
