# Supplementary material for: CD24a knockout results in an enhanced macrophage- and CD8⁺ T cell-mediated anti-tumor immune responses in tumor microenvironment in a murine triple-negative breast cancer model
Source: J Biomed Sci. 2025 Aug 9;32:73. doi: 10.1186/s12929-025-01165-3 (PMC12335121; doi:10.1186/s12929-025-01165-3)
Supplement: Supplementary file 2 — Additional file 2. [file 12929_2025_1165_MOESM2_ESM.docx]

**
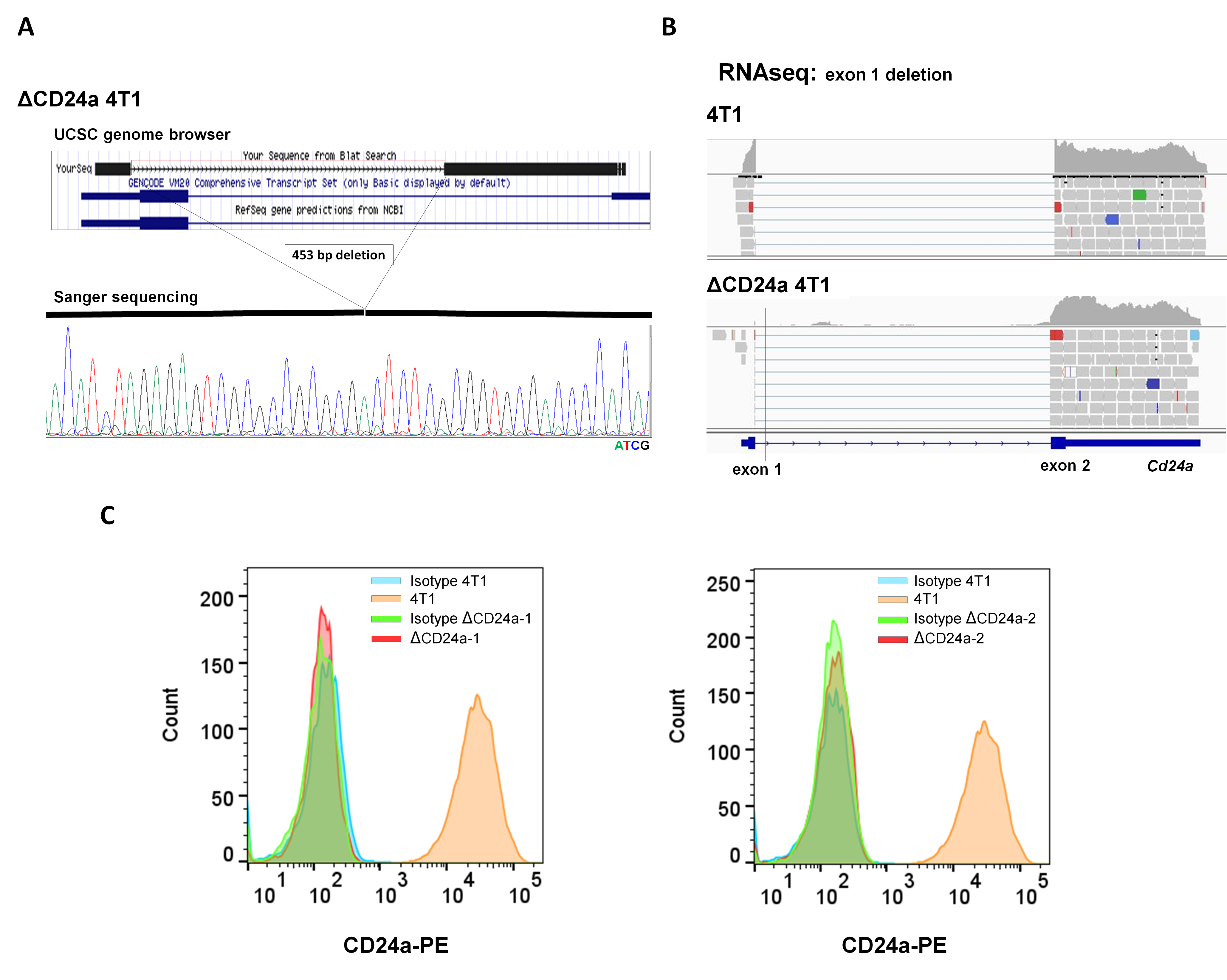
**

**Supplementary Fig. S1. CD24a expression is knocked out in 4T1 cells using a CRISPR approach. A**, Sanger sequencing of ΔCD24 4T1 cells revealed a 453 bp deletion containing exon 1 and half of intron 1 of the *Cd24a* gene, as analyzed using Blat Search in the UCSC (University of California Santa Cruz) genome browser. **B**, RNAseq analysis showed that the coding region of *Cd24a* exon 1 was deleted in ΔCD24a cells as compared to the 4T1 cells (indicated by red box). **C,** Flow cytometry analysis of CD24 expression in 4T1 cells and CD24a knockout clones (ΔCD24a-1 and ΔCD24a-2). A total of 1 × 10⁵ cells per sample were stained with a PE-conjugated anti-CD24 antibody, and fluorescence intensity (CD24-PE) was measured. The isotype control (purple line), 4T1 cells (red line) show a significant shift in fluorescence intensity, indicating high CD24 expression. In contrast, ΔCD24a-1 (orange line, left panel) and ΔCD24a-2 (green line, right panel) overlap with isotype control, confirming the absence of CD24 expression in these clones.
