# Supplementary material for: CD24a knockout results in an enhanced macrophage- and CD8⁺ T cell-mediated anti-tumor immune responses in tumor microenvironment in a murine triple-negative breast cancer model
Source: J Biomed Sci. 2025 Aug 9;32:73. doi: 10.1186/s12929-025-01165-3 (PMC12335121; doi:10.1186/s12929-025-01165-3)
Supplement: Supplementary file 3 — Additional file 3. [file 12929_2025_1165_MOESM3_ESM.docx]

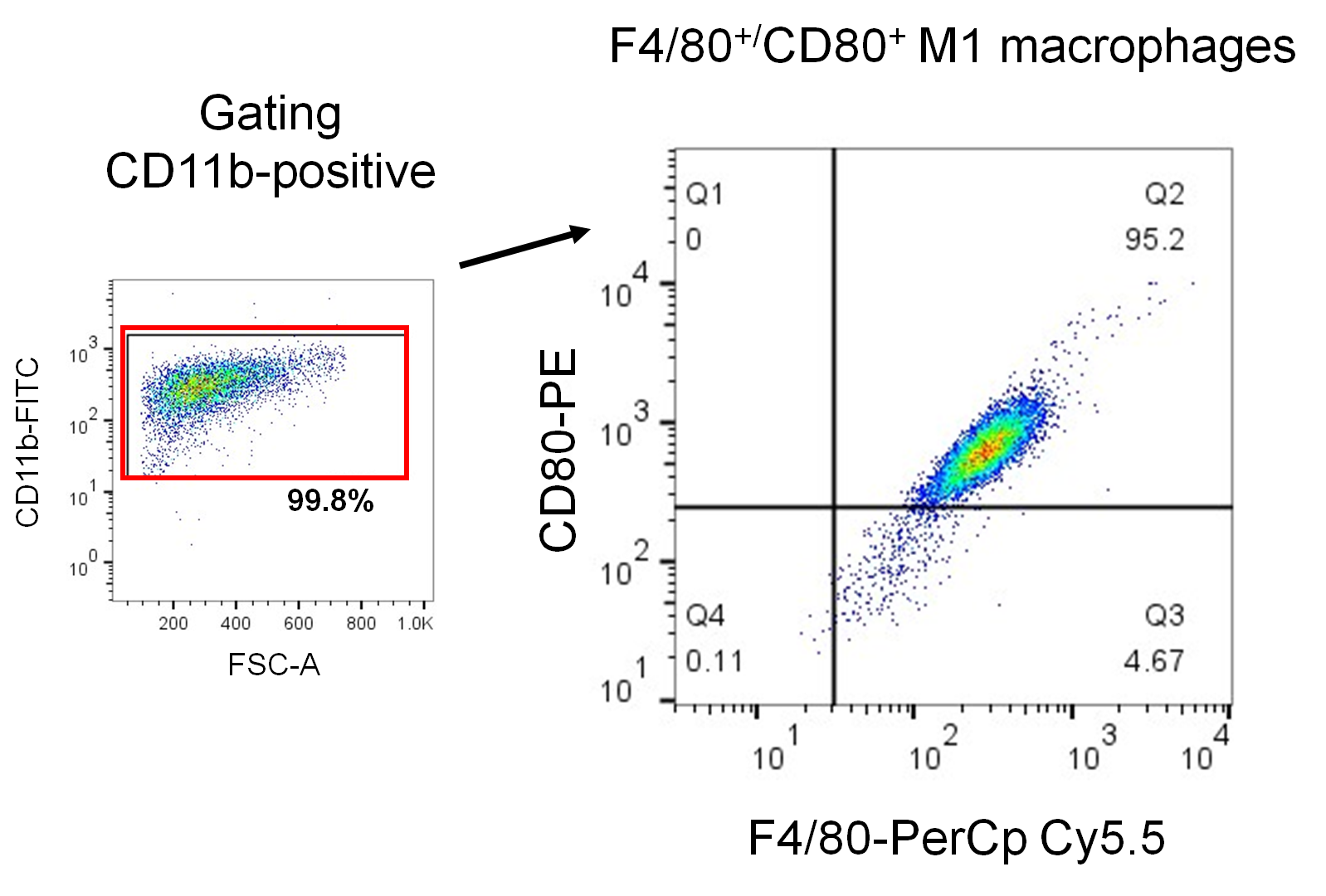


**Supplementary Fig. S2. Differentiation and characterization of CD11b^+^F4/80^+^CD80^+^ M1 macrophages from mouse bone marrow-derived macrophages.** Bone marrow cells were harvested from the femurs and tibias of euthanized mice, sterilized in 70% ethanol, and flushed with sterile PBS. The cell suspension was filtered, treated with RBC lysis buffer, and centrifuged to remove debris. Cells were cultured in RPMI-1640 complete medium supplemented containing 100 ng/mL M-CSF at 37°C with 5% CO₂. By days 7–10, adherent cells exhibited macrophage morphology and expressed F4/80 and CD11b markers. M1 polarization was achieved by stimulating BMDMs with 100 ng/mL IFN-γ for 24 hours, confirmed by upregulation of CD80 using flow cytometry. Polarized M1 macrophages were used in *in vitro* phagocytosis assay.
