# Supplementary material for: CD24a knockout results in an enhanced macrophage- and CD8⁺ T cell-mediated anti-tumor immune responses in tumor microenvironment in a murine triple-negative breast cancer model
Source: J Biomed Sci. 2025 Aug 9;32:73. doi: 10.1186/s12929-025-01165-3 (PMC12335121; doi:10.1186/s12929-025-01165-3)
Supplement: Supplementary file 4 — Additional file 4. [file 12929_2025_1165_MOESM4_ESM.docx]

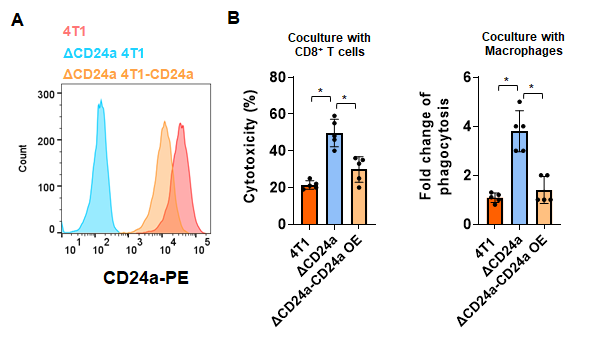


**Supplementary Fig. S3.** **CD24a deficiency significantly enhances macrophage‐mediated phagocytosis and CD8⁺ T cell-mediated cytotoxicity, effects that are partially reversed by re‐expression of CD24a. A,** Surface expression of CD24a on 4T1 (red), ΔCD24a 4T1 (blue) and CD24a-reconstituted (ΔCD24a 4T1-CD24a, orange) cells, as assessed by flow cytometry. **B,** Left: Cytotoxicity of CD8⁺ T cells against 4T1 variants was measured after 48 hours co-culture at an effector:target ratio of 20:1 using the trypan blue exclusion assay. Right: Phagocytic uptake of Calcein Am-labeled 4T1 variants by bone marrow–derived macrophages over 4 hours, quantified as fold change relative to parental 4T1 cells. Histograms are representative of three independent experiments. Data represent the mean of three independent experiments performed in triplicate. *P < 0.05 by one-way ANOVA test.
