# Supplementary material for: CD24a knockout results in an enhanced macrophage- and CD8⁺ T cell-mediated anti-tumor immune responses in tumor microenvironment in a murine triple-negative breast cancer model
Source: J Biomed Sci. 2025 Aug 9;32:73. doi: 10.1186/s12929-025-01165-3 (PMC12335121; doi:10.1186/s12929-025-01165-3)
Supplement: Supplementary file 5 — Additional file 5. [file 12929_2025_1165_MOESM5_ESM.docx]

**
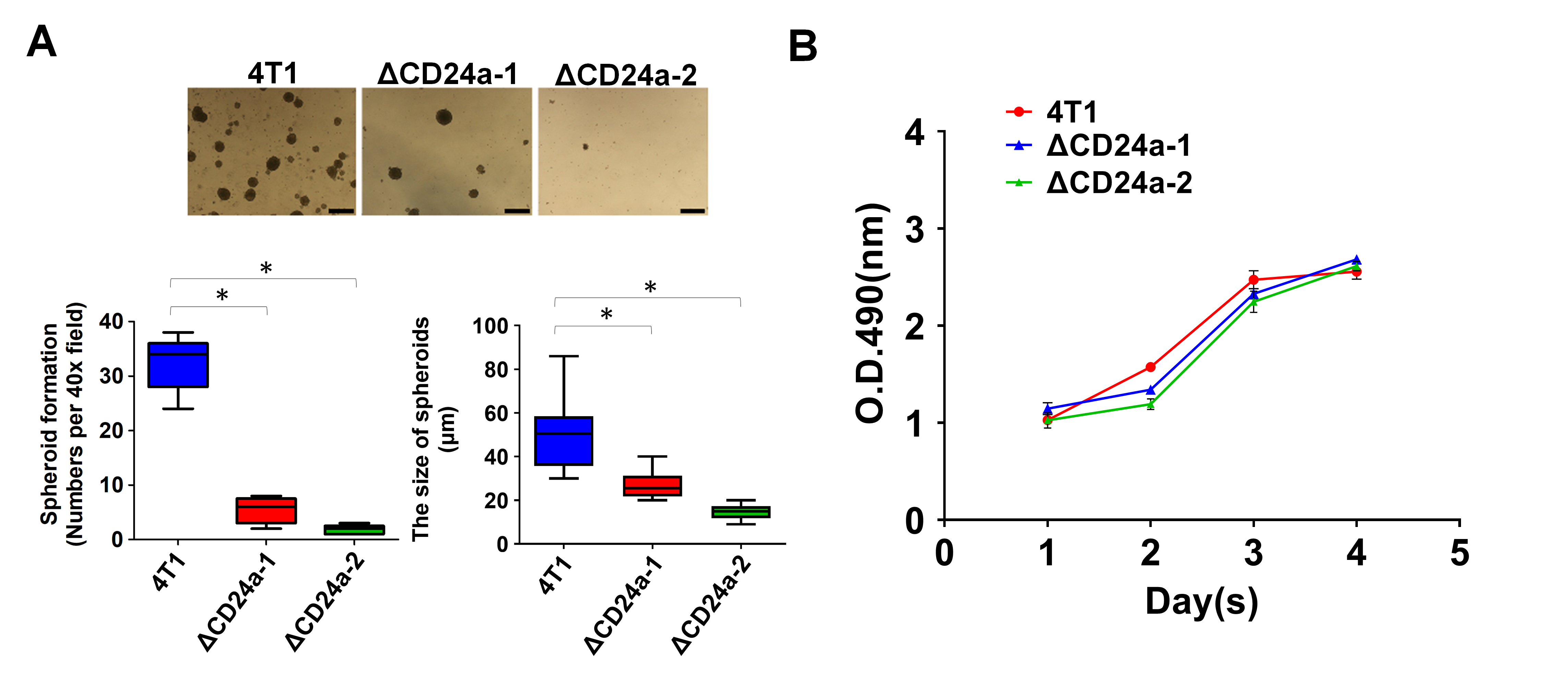
**

**Supplementary Fig. S4. CD24a knockout impairs spheroid formation of 4T1 cells without affecting cell metabolic activity. A,** Upper panel. Representative light microscopy images of tumor spheroids formed by 4T1, CD24a-1, and CD24a-2 cells cultured using the spheroid culture protocol. Scale bar: 100 μm. Lower panel. Quantification of the number and size of 4T1, CD24a-1, and CD24a-2 tumor spheroids. * P<0.01 by one-way ANOVA with Tukey’s post-hoc test. **B,** Analysis of cell metabolic of 4T1 and CD24a knockout cell using MTS assay. Data represent the mean of three independent experiments performed in triplicate.
