# Supplementary material for: CD24a knockout results in an enhanced macrophage- and CD8⁺ T cell-mediated anti-tumor immune responses in tumor microenvironment in a murine triple-negative breast cancer model
Source: J Biomed Sci. 2025 Aug 9;32:73. doi: 10.1186/s12929-025-01165-3 (PMC12335121; doi:10.1186/s12929-025-01165-3)
Supplement: Supplementary file 6 — Additional file 6. [file 12929_2025_1165_MOESM6_ESM.docx]

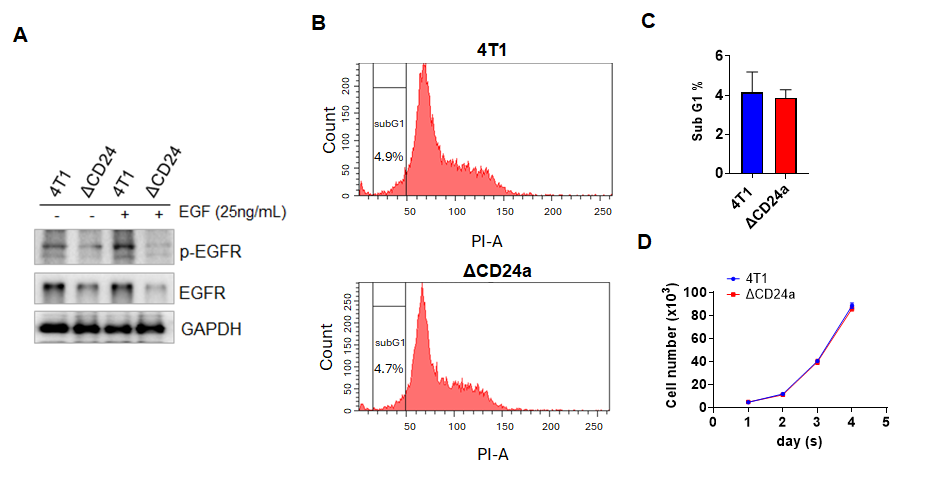


**Supplementary Fig. S5.** CD24a knockout reduces EGFR protein expression and EGF-induced EGFR phosphorylation, while cell proliferation and apoptosis rates remain unaffected. **A**, Western blot analysis of EGFR and phosphorylated EGFR (p-EGFR) in 4T1 and ΔCD24a 4T1 cells, with or without EGF (25 ng/mL) stimulation. **B,** Flow cytometry analysis of the sub-G1 phase in 4T1 and ΔCD24a 4T1 cells. **C,** Quantification of sub-G1 phase percentages in 4T1 and ΔCD24a 4T1 cells, presented as mean ± SD from three independent experiments. **D,** Cell proliferation of 4T1 and ΔCD24a 4T1 cells was assessed by measuring cell numbers over a 4-day culture period. Data represent the mean of three independent experiments performed in triplicate.
