# Supplementary material for: CD24a knockout results in an enhanced macrophage- and CD8⁺ T cell-mediated anti-tumor immune responses in tumor microenvironment in a murine triple-negative breast cancer model
Source: J Biomed Sci. 2025 Aug 9;32:73. doi: 10.1186/s12929-025-01165-3 (PMC12335121; doi:10.1186/s12929-025-01165-3)
Supplement: Supplementary file 7 — Additional file 7. [file 12929_2025_1165_MOESM7_ESM.docx]

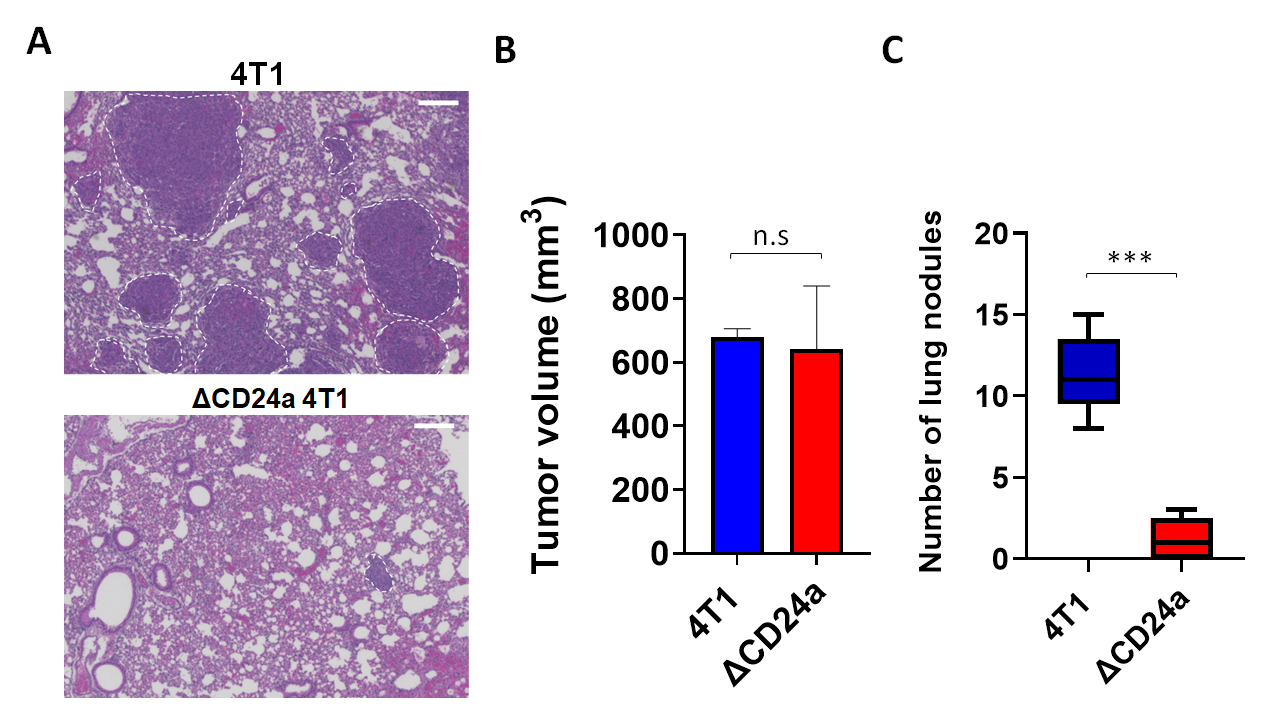


**Supplementary Fig. S6. CD24a knockout reduces lung metastasis in the 4T1 BALB/c syngeneic model. A,** Representative H&E-stained lung sections from mice bearing 4T1 or ΔCD24a 4T1 tumors. Dashed lines outline metastatic nodules. Scale bars, 200 μm. **B,** Primary tumor volumes of mice bearing 4T1 or ΔCD24a 4T1 tumors (n=3). n.s., no significant difference was observed by Student’s t-test. **C,** Quantification of lung metastatic nodules in the same cohorts (n=3). ***P < 0.001 by Student’s t-test.
