# Supplementary material for: CD24a knockout results in an enhanced macrophage- and CD8⁺ T cell-mediated anti-tumor immune responses in tumor microenvironment in a murine triple-negative breast cancer model
Source: J Biomed Sci. 2025 Aug 9;32:73. doi: 10.1186/s12929-025-01165-3 (PMC12335121; doi:10.1186/s12929-025-01165-3)
Supplement: Supplementary file 8 — Additional file 8. [file 12929_2025_1165_MOESM8_ESM.docx]

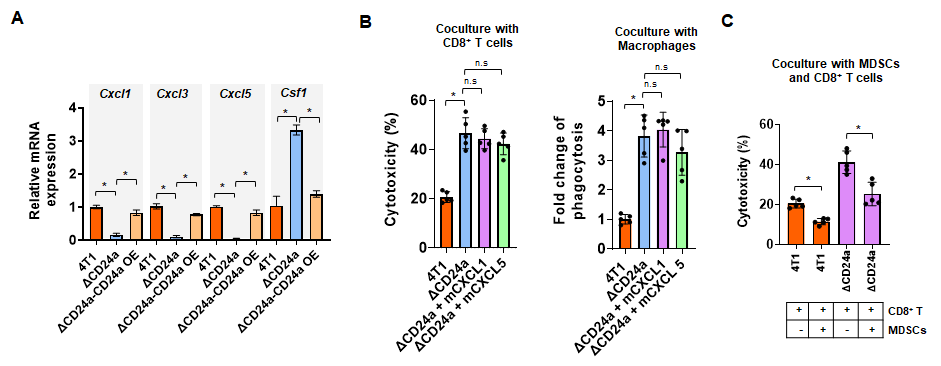


**Supplementary Fig. S7.** **Re‐expression of CD24a in CD24a Knockout 4T1 cells restores expression of gMDSC-recruiting chemokines and suppresses M-CSF. A,** qRT-PCR analysis of Cxcl1, Cxcl3 and Cxcl5 mRNA in 4T1 cells, two independent CD24a knockout clones (ΔCD24a-1, ΔCD24a-2), and CD24a-rescued cells (ΔCD24a + CD24a OE). *P < 0.05 by one-way ANOVA. **B,** Left: Cytotoxicity of murine CD8⁺ T cells against 4T1 and ΔCD24a 4T1 cells, with or without addition of recombinant murine CXCL1 or CXCL5 (100 ng/mL), measured after 24 h at a 20:1 effector:target ratio (*P < 0.05). Right: Fold-change in phagocytic uptake of Calcein AM-labeled 4T1 variants by bone marrow–derived macrophages under the same conditions (*P < 0.05) . Data represent the mean of three independent experiments performed in triplicate. *P < 0.05.
