# Supplementary material for: CD24a knockout results in an enhanced macrophage- and CD8⁺ T cell-mediated anti-tumor immune responses in tumor microenvironment in a murine triple-negative breast cancer model
Source: J Biomed Sci. 2025 Aug 9;32:73. doi: 10.1186/s12929-025-01165-3 (PMC12335121; doi:10.1186/s12929-025-01165-3)
Supplement: Supplementary file 9 — Additional file 9. [file 12929_2025_1165_MOESM9_ESM.docx]

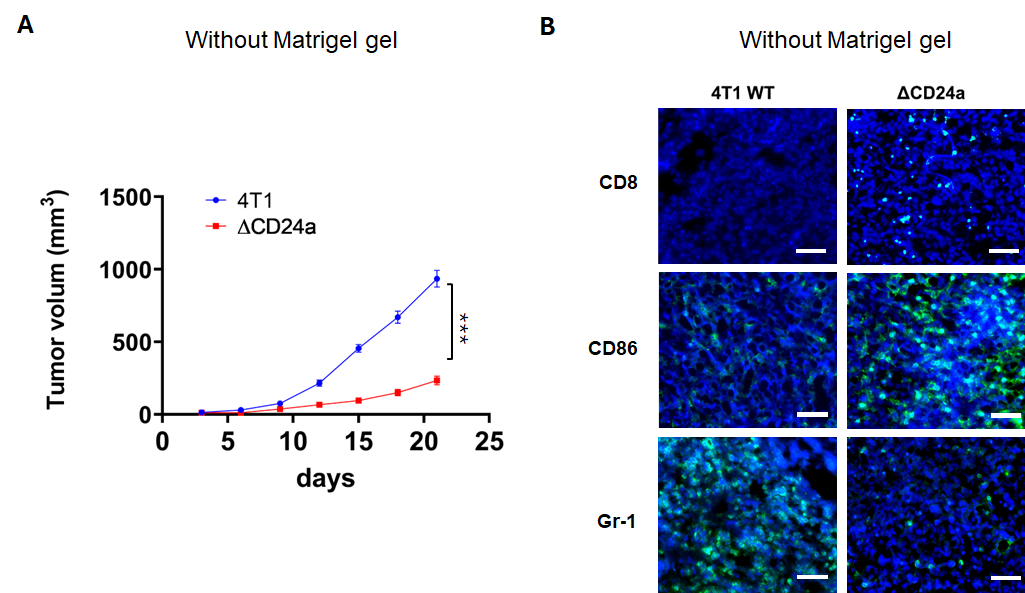


**Supplementary Fig. S8. CD24a knockout delays tumor growth and enhances immune infiltration in a Matrigel-free orthotopic 4T1 BALB/c model. A,** Tumor growth kinetics in BALB/c mice revealed that ΔCD24a 4T1 cells, implanted without Matrigel, exhibited significantly delayed growth compared to 4T1 cells. 5 x 10^5^ 4T1 or ΔCD24a 4T1 cells were orthotopically implanted in BALB/c mice, respectively (n=3), and tumor growth of mice was monitored until day 21 post-inoculation. ***P<0.001. **B**, Representative IF images of tumor sections showed increased infiltration of CD86⁺ M1 macrophages and CD8^+^ T cells, with decreased Gr-1⁺ MDSCs, in the TME of ΔCD24a 4T1 tumors compared to 4T1 tumors. Scale bar, 50 μm**.**
